# ADAM22/LGI1 complex as a new actionable target for breast cancer brain metastasis

**DOI:** 10.1186/s12916-020-01806-4

**Published:** 2020-11-19

**Authors:** Sara Charmsaz, Ben Doherty, Sinéad Cocchiglia, Damir Varešlija, Attilio Marino, Nicola Cosgrove, Ricardo Marques, Nolan Priedigkeit, Siobhan Purcell, Fiona Bane, Jarlath Bolger, Christopher Byrne, Philip J. O’Halloran, Francesca Brett, Katherine Sheehan, Kieran Brennan, Ann M. Hopkins, Stephen Keelan, Petra Jagust, Stephen Madden, Chiara Martinelli, Matteo Battaglini, Steffi Oesterreich, Adrian V. Lee, Gianni Ciofani, Arnold D. K. Hill, Leonie S. Young

**Affiliations:** 1 Endocrine Oncology Research Group, Department of Surgery, Royal College of Surgeons in Ireland, Dublin 2, Ireland; 2 Smart Bio-Interfaces, Istituto Italiano di Tecnologia, Scuola Superiore Sant’Anna, Pontedera, Italy; 3 Women’s Cancer Research Centre, Magee-Women’s Research Institute, University of Pittsburgh Cancer Institute, University of Pittsburgh, Pittsburgh, PA USA; 4 Department of Neurosurgery, National Neurosurgical Centre, Beaumont Hospital, Dublin, Ireland; 5 Department of Neuropathology, Beaumont Hospital, Dublin, Ireland; 6 Department of Pathology, Royal College of Surgeons in Ireland, Dublin, Ireland; 7 Department of Surgery, Royal College of Surgeons in Ireland, Dublin, Ireland; 8 Data Science Centre, Royal College of Surgeons in Ireland, Dublin, Ireland; 9 The Biorobotics Institute, Scuola Superiore Sant’Anna, Pontedera, Italy; 10 Department of Surgery, Beaumont Hospital, Dublin, Ireland

**Keywords:** Breast cancer metastases, Brain metastases, ADAM22, LGI1, ECM signalling, Blood–brain barrier, Targeted therapy

## Abstract

**Background:**

Metastatic breast cancer is a major cause of cancer-related deaths in woman. Brain metastasis is a common and devastating site of relapse for several breast cancer molecular subtypes, including oestrogen receptor-positive disease, with life expectancy of less than a year. While efforts have been devoted to developing therapeutics for extra-cranial metastasis, drug penetration of blood–brain barrier (BBB) remains a major clinical challenge. Defining molecular alterations in breast cancer brain metastasis enables the identification of novel actionable targets.

**Methods:**

Global transcriptomic analysis of matched primary and metastatic patient tumours (*n* = 35 patients, 70 tumour samples) identified a putative new actionable target for advanced breast cancer which was further validated in vivo and in breast cancer patient tumour tissue (*n* = 843 patients). A peptide mimetic of the target’s natural ligand was designed in silico and its efficacy assessed in in vitro, ex vivo and in vivo models of breast cancer metastasis.

**Results:**

Bioinformatic analysis of over-represented pathways in metastatic breast cancer identified ADAM22 as a top ranked member of the ECM-related druggable genome specific to brain metastases. ADAM22 was validated as an actionable target in in vitro, ex vivo and in patient tumour tissue (*n* = 843 patients). A peptide mimetic of the ADAM22 ligand LGI1, LGI1MIM, was designed in silico*.* The efficacy of LGI1MIM and its ability to penetrate the BBB were assessed in vitro, ex vivo and in brain metastasis BBB 3D biometric biohybrid models, respectively. Treatment with LGI1MIM in vivo inhibited disease progression, in particular the development of brain metastasis.

**Conclusion:**

ADAM22 expression in advanced breast cancer supports development of breast cancer brain metastasis. Targeting ADAM22 with a peptide mimetic LGI1MIM represents a new therapeutic option to treat metastatic brain disease.

## Background

Breast cancer metastasis is responsible for the vast majority of all breast cancer related deaths [1]. Although overall clinical molecular subtype switching on metastasis is rare, global alterations in the transcriptome are known to facilitate disease progression. Longitudinal sequencing studies of matched primary and metastatic tumours define transcriptional gains in tyrosine kinase and extracellular matrix (ECM)/cell-to-cell communication signalling networks [2, 3], which may represent acquired vulnerabilities and open up new therapeutic opportunities. A disintegrin and metalloproteinases (ADAMs) and the related ADAM with thrombospondin motifs (ADAMTS) are key members of the ECM signalling network [4, 5]. ADAMs are a family of multi-domain transmembrane proteins [6, 7], with functions in cell adhesion, migration and proteolysis [8]. Approximately half the family members have matrix metalloproteinase-like activity, with several members, including ADAM10 and 17 being targeted for cancer therapy [9].

We have previously demonstrated a role for ADAM22 in endocrine-resistant breast cancer and identified ADAM22 as an independent predictor of poor disease-free survival [10]. Furthermore, recent studies by Li et al. [11] reported upregulation of ADAM22 through decreased miR-449a, resulting in the development of tamoxifen resistance in ER-positive breast cancer cells. These studies suggest a role for ADAM22 in the development of endocrine-resistant disease and metastatic progression [10]. Unlike other family members implicated in cancer, ADAM22 lacks a functional metalloproteinase domain [12] and may mediate its pro-tumourigenic effects through interaction with other cell surface tyrosine kinase receptors using its EGF-like domain [6].

At a physiological level, ADAM22 acts as a receptor on the surface of the postsynaptic neuron to regulate signal transmission through binding to leucine-rich, glioma inactivated gene 1 (LGI1), a neuronal protein and a specific ligand for ADAM22 [12]. The LGI1/ADAM22 complex has been reported to have a role in epilepsy, and the ligand/receptor has been suggested as a therapeutic target for synaptic disorders [13]. In cancer, LGI1 has been shown to function as a tumour suppressor gene for glioblastoma and neuroblastoma [14, 15], and we have previously reported LGI1-targeted inhibition of cell migration in ER-positive endocrine-resistant breast cancer cells mediating distant metastatic competency [10].

Breast cancer is the second most common primary tumour type that spreads to the brain and affects approximately 10% of ER-positive breast cancer patients [16]. Metastasis to the brain confers particularly poor prognosis with life expectancy following diagnosis of less than a year. Currently, the standard therapeutic for the vast majority of breast cancer brain metastasis patients includes surgery, radiotherapy and in some cases systemic chemotherapy, all which have limited success [16]. Identifying new therapeutics for breast cancer brain metastasis, which can target brain colonising cells and can cross the blood–brain barrier, is required. Here we investigate the role of ADAM22 in ER-positive breast cancer brain metastasis and evaluate the potential of targeting ADAM22 with the small peptide mimetic of LGI1 as a new therapeutic strategy to treat brain metastasis.

## Methods

### Ethics

All clinical material was collected under the clinical trial NCT01840293 (https://clinicaltrials.gov) following ethical approval from Beaumont Hospital Medical Research Ethics Committee.

### Cell culture

The MCF7 cells were obtained from ATCC and cultured in Minimum Essential Medium Eagle (MEM) (M2279, Sigma) supplemented with 2 mM l-glutamine (G7513, Sigma) and 10% fetal calf serum (FCS) (F7524, Sigma). The LY2 cells were a gift from Robert Clarke (Georgetown, USA) and were cultured as previously described [17]. Each cell line was tested for mycoplasma contamination (LT07-118, Lonza) and genotyped (Source BioScience) according to ATCC guidelines. T347 cells were derived from an ER+ PR− HER2+ brain metastatic tumour from a breast cancer patient as previously described [18]. AI resistant LetR cells were generated as previously described [19].

### Bioinformatics

#### Differential gene expression—exome capture RNA sequencing of brain and bone metastasis

Sequencing reads from previously published exome capture sequencing of patient-matched primary breast tumour with bone metastases [20] and brain metastases [3] were mapped against human reference transcriptome GRCh38.p10 using Salmon v.0.9.1 [21]. Gene level summarised counts were used as input for differential gene expression. *DESeq2* [22] was used to identify differential gene expression separately in primary breast tumours compared to matched brain metastases and matched bone metastases. A multi-factor design was used for matched (gene_i_ ~ patient + tumour). A log2 fold change of greater than ± 1.5 and an adjusted *p* value of < 0.05 were used to identify up- or downregulated genes. *EnhancedVolcano* was used to generate volcano plots of differential gene expression [23], with genes upregulated in metastasis annotated by Matrisome database version 2.0 [23] labelled.

#### Differential gene expression—RNA sequencing of liver metastasis

Raw sequencing reads [2] were processed using *fastQC*, *bbmap* and *subreads* to obtain normalised gene counts for 3 patient-matched primary breast with liver metastases including 2 matched nodal samples. Differential gene expression testing was performed using *edgeR* [24] (log2 fold change ± 1.5 FDR < 0.05).

#### Gene Ontology Enrichment Testing

The *clusterProfiler* package in R version 3.5.2 was used to functionally annotate genes according to Gene Ontology biological process (BP) categories using gene symbol ids as input [25]. Statistically significant differential genes identified from breast cancer brain, bone and liver metastases were treated as individual gene clusters for *CompareCluster* function. The hypergeometric test was used with significant GO terms for each cluster called based on a *q*-value < 0.05. Significant GO terms were visualised using the *dotplot* function.

#### Gene expression gains in druggable genome

*DESeq2* [22] was used to identify differential gene expression in ER-positive primary breast tumours compared to matched brain metastases using salmon gene counts from a previously published dataset [3]. A multi-factor design was used for matched samples (gene_i_ ~ patient + tumour). A log2 fold change of greater than ± 1.5 and an adjusted *p* value of < 0.05 were used to identify up- or downregulated genes. In order to identify extracellular matrix (ECM)-related genes upregulated in ER-positive primary breast tumours versus brain metastases, genes were cross referenced against the Matrisome database version 2.0 [23]. Druggable genome categorised genes (*n* = 6106) were downloaded from the Drug-Gene Interaction database version 3.0 (DGIdb 3.0) [26]. ECM-related genes were further cross-referenced against “druggable genome” genes. Identification of recurrent gene expression gains were performed as per Vareslija et al. [3]. After assignment of discrete expression gains in ECM druggable genome genes, those genes with gains in greater than 3 patients were plotted using the *oncoprint* function in *ComplexHeatmap* [27].

#### Association of ADAM22 with brain predominant marker

A gene–gene Pearson correlation coefficient for known brain predominant genes [28, 29] and ADAM22 in breast cancer brain metastatic tumours (*N* = 21 patients) was undertaken. Correlation score of *p* < 0.05 was considered significantly correlated.

### CRISPR/Cas9 and lentiviral transduction

Full details can be found in supplementary methods (Additional file 1). Briefly, ADAM22 was knocked out in LY2 cells using CRISPR/Cas9 technology (Additional file 2 Fig. S1 a-e) and ADAM22 was over-expressed with lentiviral particles (Additional file 2 Fig. S2 a-d).

### Mammosphere forming, anchorage independence and cell migration assays

Functional assays were performed in the MCF7, LY2, LY2 ADAM22 KO, LY2 ADAM22 KI, LETR and T347 cells and were carried out in the presence of 4-OHT [10^− 7^ M] where appropriate. Mammosphere culture and analysis was performed as previously described [30]. Anchorage independence was analysed using the agarose colony formation assay as previously described [31]. Cell migration was carried out using the Cellomics Cell Motility Kit (K0800011, Thermo Scientific) as previously described [32].

### Reverse phase proteomic study (RPPA)

RPPA analysis was carried out on protein lysates from LY2 and LY2 ADAM22 KO using a Genetix QArray2 spotter (Additional file 2 Fig. S3a). Full details can be found in Supplementary methods.

### Immunohistochemistry (IHC)

ADAM22 expression was examined on a tissue microarray (TMA) of breast cancer samples in a cohort of patients diagnosed from 2008 to 2015 from Beaumont Hospital, Dublin (*n* = 843) as previously described [33]. The TMA was immunostained using C-2 monoclonal ADAM22 antibody (sc-373,931, Santa Cruz) (8 μg/ml) and scored using the histoscore method. Matched primary and metastatic tissues were from patients from the above cohort, and healthy normal tissue sections from the brain, lung, liver, heart, spleen and kidney were obtained from Beaumont Hospital and scored using the histoscore method.

### Peptide synthesis

Computer modelling was used to model ADAM22/LGI1MIM interactions; details are provided in Supplementary methods. A 21 a.a. peptide mimetic of the ADAM22 disintegrin binding domain of LGI1 (LGI1MIM) was designed using I-TASSER software with a single cysteine to serine substitution (a.a. 447). Full length LGI1 and the peptide mimetic LGI1 was synthesised to > 95% purity by JPT Peptide Technology, Berlin.

### Organoid culture

Organoid culture was generated from brain metastatic breast patient-derived sample (T347) following Sachs et al.’s protocol [34]. Briefly, frozen pieces (3–6 mm^3^) were minced, enzymatically digested with collagenase (1 mg/ml, Sigma, C9407) for 1.5 h at 37 °C followed by mechanical digestion. Ten thousand single cells (70 μm strained) were seeded in organoid media [34] with 5% of Cultrex® Reduced Growth Factor Basement Membrane Matrix, type 2 (BME, Trevigen, 3533-001-02). Once formed, organoids were treated with vehicle (0.1% DMSO) or LGI1 (25 nM). Each treatment was tested in multiple replicates (> 5). Cell viability was measured 7 days post treatment using CellTiter-Glo® 3D Cell Viability assay (Promega) as per the manufacturer’s protocol.

### Ex vivo assay and Ki67 staining

Patient breast cancer brain metastatic ER-positive tumours (T347, T2447 and T328) and triple-negative tumour (T2203) were expanded in NOD/SCID mice [3]. The primary tumours were resected, grown on gelatin sponges (Spongostan, Johnson and Johnson) as previously described [33] and treated with oestrogen in the presence of LGI1 peptide mimetic (LGI1MIM) or vehicle for 72 h. Following treatment, tumour pieces were formalin fixed and paraffin embedded for IHC analysis. The viability of the tumours was evaluated by screening for necrosis of the tissue and assessed for Ki67 by IHC to confirm viable, proliferating cells.

### Statistics

Statistical analysis was performed using Prism (GraphPad, San Diego, CA, USA) and Stata software (StataCorp, College Station, TX, USA). Survival times between groups were calculated using log rank for quality of survival and compared with a *χ*^2^ distribution with 1 degree of freedom. Multivariate analysis for ADAM22 was carried out using Cox proportional hazards, modelled with PR status, HER2 status and node using the Breslow method for ties. Other appropriate statistical tests for each experiment were undertaken as described in the relevant “Results” section. Values of *p* ≤ 0.05 were considered significant.

### In vivo experiments

All animal experiments were performed in accordance with the European Communities Council Directive (2010/63/EU) and were reviewed and approved by Research Ethics Committee, RCSI under licence from the Department of Health and The Health Products Regulatory Authority (SI No543 2012). Luciferase-tagged, LY2, LY2 ADAM22 KO cells and LY2 ADAM22 KI cells (Additional file 2 Fig. S3 b-d) were injected into the mammary fat-pad of NOD/SCID mice. Full details are available in Supplementary methods.

### Biomimetic LGI1MIM-loaded liposome (LGI1MIM-LS) preparation, functionalisation and staining

LGI1MIM-LSs were obtained using the thin-film evaporation method as described in Supplementary methods. LGI1MIM-LSs were functionalised with a streptavidine-conjugated anti-transferrin receptor antibody (anti-TfR; 25 μg/ml) and purified [35]. For the evaluation of blood–brain barrier (BBB) crossing, 5 mg/ml of LSs were stained with Vybrant™ DiO labelling solution (1:25 dilution) and purified. LGI1MIM-LSs are characterised by unilamellar morphology and homogeneous size distribution of 100 nm diameter (Additional file 2 Fig. S4a).

### LGI1MIM-LS imaging and characterisation

Transmission electron microscopy (TEM) analyses were carried out with a transmission electron microscope (JEOL 1011, Tokyo, JAPAN). Size and Z-potential distributions of a 100 μg/ml LGI1MIM-LSs dispersion were analysed using a Zetasizer Nano ZSP (Malvern Instrument). Full details are available in Supplementary methods.

### Multicellular blood–brain barrier (BBB) in vitro model

BBB model was obtained by culturing the brain-derived endothelioma bEnd.3 cells (ATCC® CRL-2299™ at 8 × 10^4^ cells/cm^2^) and the C8D1A brain astrocytes (ATCC® CRL-2541™ at 2 × 10^4^ cells/cm^2^) on the luminal and abluminal side of 3 μm porous transwells (Corning Incorporated). Expression of tight junction proteins and BBB crossing were assessed as described in Supplementary methods.

### Evaluation of LGI1MIM-LSs internalisation, Ki-67 expression and cell cycle analysis in T347 cells

Abluminal compartments were transferred to 24-well plates where T347 cells were seeded 24 h prior to the start of the experiment. 500 μg/ml LGI1MIM-LSs was added to the luminal compartment and incubated for 72 h (Additional file 2 Fig. S4). Liposome uptake, cell viability, Ki-67 expression and cell cycle analysis were investigated in the T347 cells cultured in the abluminal compartment. 3D CLSM of DiO-stained LGI1MIM-LSs internalised in T347 cells was performed using a C2s system (Nikon).

### WST-1 and Ki67 proliferation assays

Proliferation assays were carried out ex vivo on brain metastasis tissue, in vitro on T347 cells and on the T347 cells in the BBB model system. Cell proliferation was assessed using a standard WST-1 assay. Ki-67 was assessed by IHC with mouse monoclonal anti-Ki67 (DAK0) and peroxidase-based EnVision+ kit (DAKO) (ex vivo tissue) and a TRITC-conjugated secondary antibody (Millipore). Both assays are described in detail in the supplemental methods.

## Results

### ECM is a key pathway in breast cancer metastasis and the ECM signalling protein ADAM22 promotes distant metastatic disease burden in vivo

**Fig. 1:**
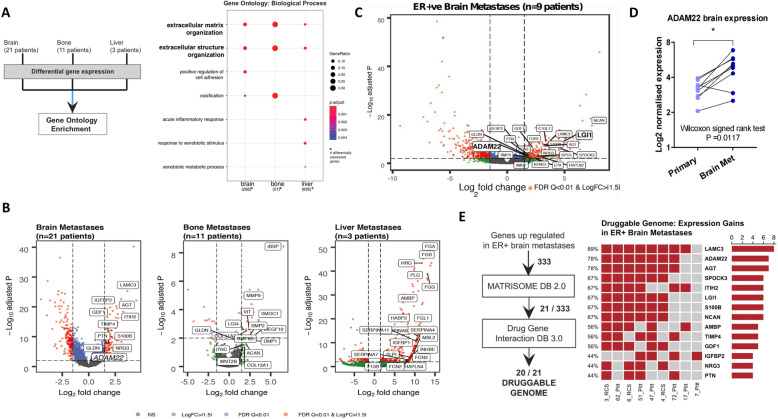
ECM is a key pathway in breast cancer metastasis and the ECM signalling protein ADAM22 promotes distant metastatic disease burden in vivo. **a** Sequencing reads from exome capture RNAseq of patient-matched primary breast tumour with bone metastases (*n* = 11 patients) and brain metastases (*n* = 21 patients) and from RNA seq of matched primary and metastatic liver (*n* = 3 patients) were mapped against human reference transcriptome GRCh38.p10. *DESeq2* (exome capture RNAseq) and *edgeR* (RNAseq) were used to identify differential gene expression separately in primary breast tumours compared to matched brain (266), bone (51) and liver (695) metastases (log2 fold change ± 1.5 FDR < 0.05). Dotplot (right) of functional annotation of differentially expressed genes using gene ontology biological process (hypergeometric test *q*-value < 0.05). **b** Differentially expressed genes (log2 FC > 1.5, adjusted *p* value < 0.01) are displayed in the volcano plot. Genes were cross referenced against the Matrisome database to identify extracellular matrix (ECM) related genes (labelled in the volcano plot). **c** Exome-capture RNAseq of ER-positive primary breast and matched brain metastatic tissues (*n* = 9 patients, 18 samples). Differentially expressed genes (log2 FC > 1.5, adjusted *p* value < 0.01) are displayed in the volcano plot. Genes were cross referenced against the Matrisome database to identify extracellular matrix (ECM)-related genes (labelled in the volcano plot). **d** A significant increase in ADAM22 expression was found in brain metastases in patients in comparison to matched primary tissue, *n* = 9, Wilcoxon matched-pairs signed-rank test *p = 0.0117. **e** Oncoprint of recurrent (> 3 patients) Matrisome and Druggable Genome (Drug-Gene Interaction Database)-related gene expression gains in ER-positive brain metastases patients (*n* = 9) showed ADAM22 (78%) as the second-ranked druggable genome in the list

RNA-seq cohorts of patient-matched primary and metastatic bone (*n* = 11 patients), brain (*n* = 21 patients) and liver (*n* = 3 patients) were investigated for metastasis-specific alterations. Differential gene expression functionally annotated using gene ontology demonstrated a significant enrichment for ECM and extracellular structure organisation function to all three sites (Fig. 1a, Additional file 3 Table S1). Volcano plots of differentially expressed genes were annotated by Matrisome database to further understand aberrant ECM signalling in metastasis, which confirmed the metalloproteinase family member, ADAM22, as elevated in brain metastatic tumours (Fig. 1b, Additional file 3 Table S1).

We have previously demonstrated the role for ADAM22 in ER-positive breast cancer [10]. Here we have further analysed the exome-capture RNA sequencing filtered through the Matrisome database in ER-positive patients (*n* = 9) and identified elevation in ADAM22 expression in breast cancer brain metastatic tumours (Fig. 1c, Additional file 4 Table S2), with significant gain of ADAM22 expression in ER-positive brain metastasis (*n* = 9 patients, *p = 0.0117*) (Fig. 1d). Moreover, ADAM22 expression correlated with known brain metastatic associated [28, 29] in both primary (MOCS1, *n* = 21, *p* < 0.05) and metastatic tumours (MOCS1, COL13A1, TLR4, HBEGF and PELI2, *n* = 21, *p* < 0.05).

Twenty of the identified ECM-related upregulated genes in ER-positive primary versus brain metastases were classified as druggable genome targets in the Drug Gene Interaction database. ADAM22 was a top ranked druggable genome target showing recurrent (*n* > 3 patients) gene expression gains in ER-positive brain metastatic patients (Fig. 1e, Additional files 4 and 5 Table S2 and S3). These data raise the potential of ADAM22 as a therapeutic target in advanced ER-positive breast cancer.

### ADAM22 expression promotes metastatic characteristics in vivo and in vitro

**Fig. 2:**
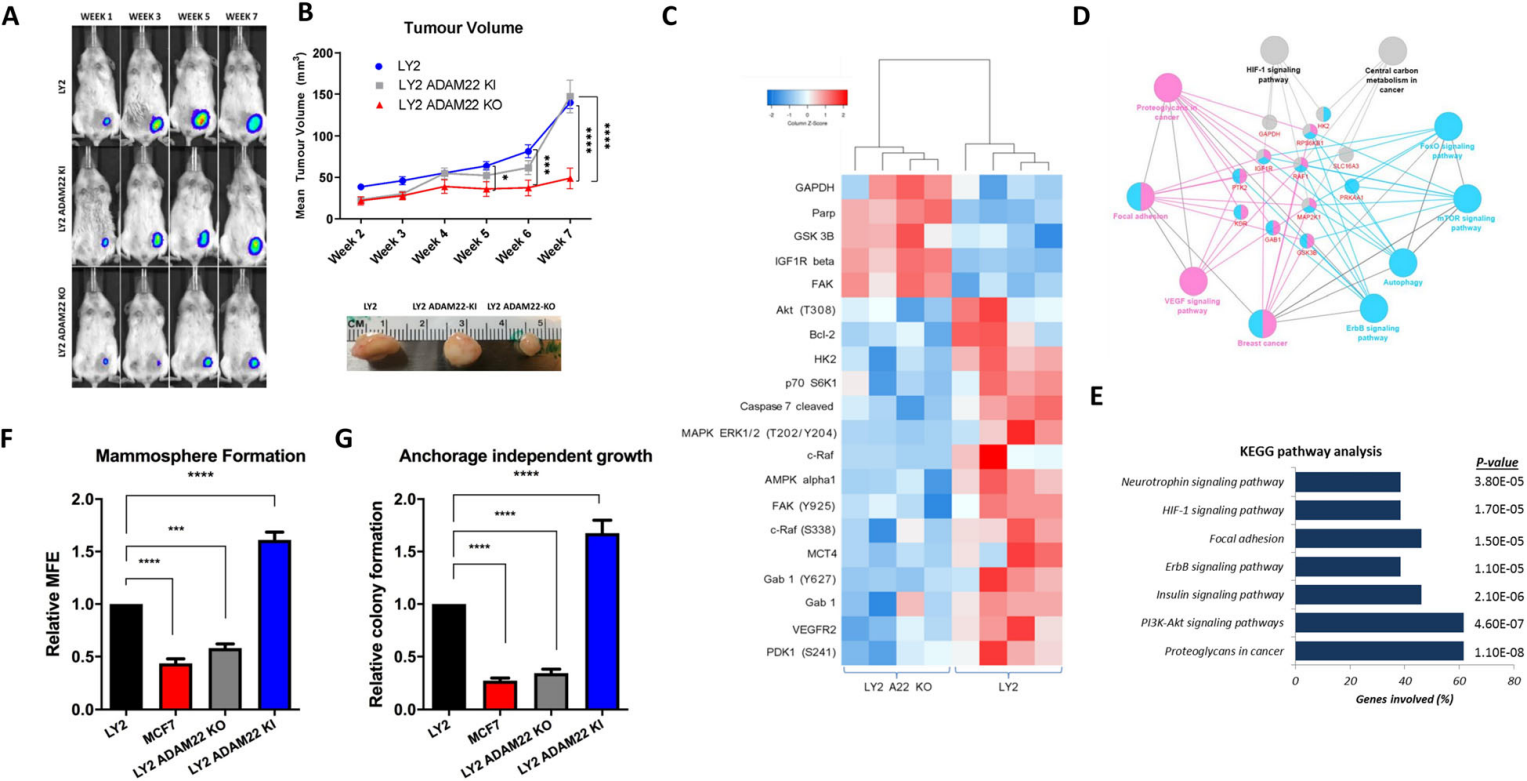
ADAM22 expression promotes metastatic potential of endocrine resistant cells via upregulation of pro-metastatic signalling pathways. **a** LY2 WT, LY2 ADAM22 KI and LY2 ADAM22 KO (1 × 10^6^) cells were luciferase tagged and injected into the mammary fat pad of NOD/SCID mice (*n* = 8, *n* = 7 and *n* = 7, respectively) and allowed to grow to approximately 150 mm^3^. **b** Tumour growth was assessed by IVIS 15 min post luciferin injection (150 μg/g). Tumour volume was measured by weekly calliper measurements using the following formula: Volume mm^3^ = (length × (smallest width) × 2) × 0.5. Statistical significance was calculated using 2-way ANOVA multiple comparison test *p = 0.0395, ***p < 0.0005, ****p < 0.0001. **c** Heatmap from RPPA analysis showing proteins/phospho-proteins differentially expressed in LY2 and LY2 ADAM22-KO cells following 4-OHT (10^− 7^ M) treatment for 15 min (LIMMA adjusted *p* value < 0.05; red = upregulated; blue = downregulated). **d** Pathway analysis using ClueGo represent the common pathways from KEGG pathway analysis using the common targets from 4 or more differentially expressed proteins by ADAM22. **e** Top ranking KEGG pathways associated with ADAM22 dependent 4-OHT response. **f** ADAM22 promotes mammosphere formation. LY2, MCF7, LY2 ADAM22 KO and LY2 ADAM22 KI cells were plated in mammosphere-forming medium supplemented with 4-OHT (10^− 8^ M) for 5 days. Mammospheres (> 50 μm) were counted to determine the mammosphere forming efficiency (MFE). Bar graphs show relative (to LY2) MFE ± SEM from three independent experiments. Statistical significance was calculated using one-way ANOVA, ***p = 0.0001 ****p < 0.0001. **g** ADAM22 expression promotes anchorage independent colony formation. LY2, LY2 ADAM22 KO, MCF7 and LY2 ADAM22 KI cells cultured in an anchorage independent state for 14 days. Colonies were stained with p-iodonitrotetrazolium and counted. Bar graphs show relative (to LY2) colony formation ± SEM from three independent experiments. Statistical significance was calculated using one-way ANOVA, ****p < 0.0001

Here we examined the ability of ADAM22 to drive disease progression in an in vivo model of endocrine-resistant breast cancer. Tamoxifen-resistant LY2 wild-type (WT), LY2 ADAM22 overexpressing (LY2 ADAM22 KI) and ADAM22 CRISPR/Cas9 knock-out cells (LY2 ADAM22 KO) were injected into the mammary fat pad of the NOD/SCID mice to determine the effect of ADAM22 on breast cancer disease progression in vivo (Fig. 2a)*.* Mice implanted with ADAM22 KO cells had significantly reduced tumour volume in comparison to mice implanted with endogenous ADAM22 or ADAM22 KI cells (*p* < 0.0001 and *p* = 0.0032, respectively) (Fig. 2b). Furthermore, decreased tumour weight was observed between ADAM22 KO and WT tumours (*p* < 0.05) (Additional file 2 Fig. S3d).

Given the role of ADAM22 in breast cancer progression, the mechanism of action of the ADAM22 protein was investigated. Reverse phase protein array (RPPA) was used to explore ADAM22-dependent protein signalling in LY2 and LY2 ADAM22 KO cells in the presence of tamoxifen (4-OHT). A total of 20 proteins and phospho-proteins were found to be differentially expressed (Fig. 2c). Functional network analysis using ClueGo identified pro-proliferative and pro-metastatic pathways associated with ADAM22 expression in endocrine resistance including growth factor signalling (ERBB, VEGF and IGFR) and cell to cell communication (focal adhesion) (Fig. 2d and e). Top ranking KEGG pathways included PI3-AKT, neurotrophin signalling, focal adhesion and ERBB signalling (*p* < 4.60E−07, 3.80E−5, 1.50E−05 and 1.10E−05, respectively) (Fig. 2e). The functional consequences of activation of these pathways were evidenced by greater mammosphere formation and anchorage independent growth in endocrine-resistant LY2 cells in comparison to LY2 ADAM22 KO and endocrine sensitive MCF7 cells (*p* < 0.0001) (Fig. 2f and g). Moreover, elevations in mammosphere formation and anchorage independent growth were observed in LY2 ADAM22 KI cells in comparison to wild-type ADAM22 cells consistent with the characteristics of stemness and tumourigenic potential (*p* = 0.0002 and *p* = 0.0012, respectively) (Fig. 2f and g).

### ADAM22 is a promising drug target for metastatic breast cancer

**Fig. 3:**
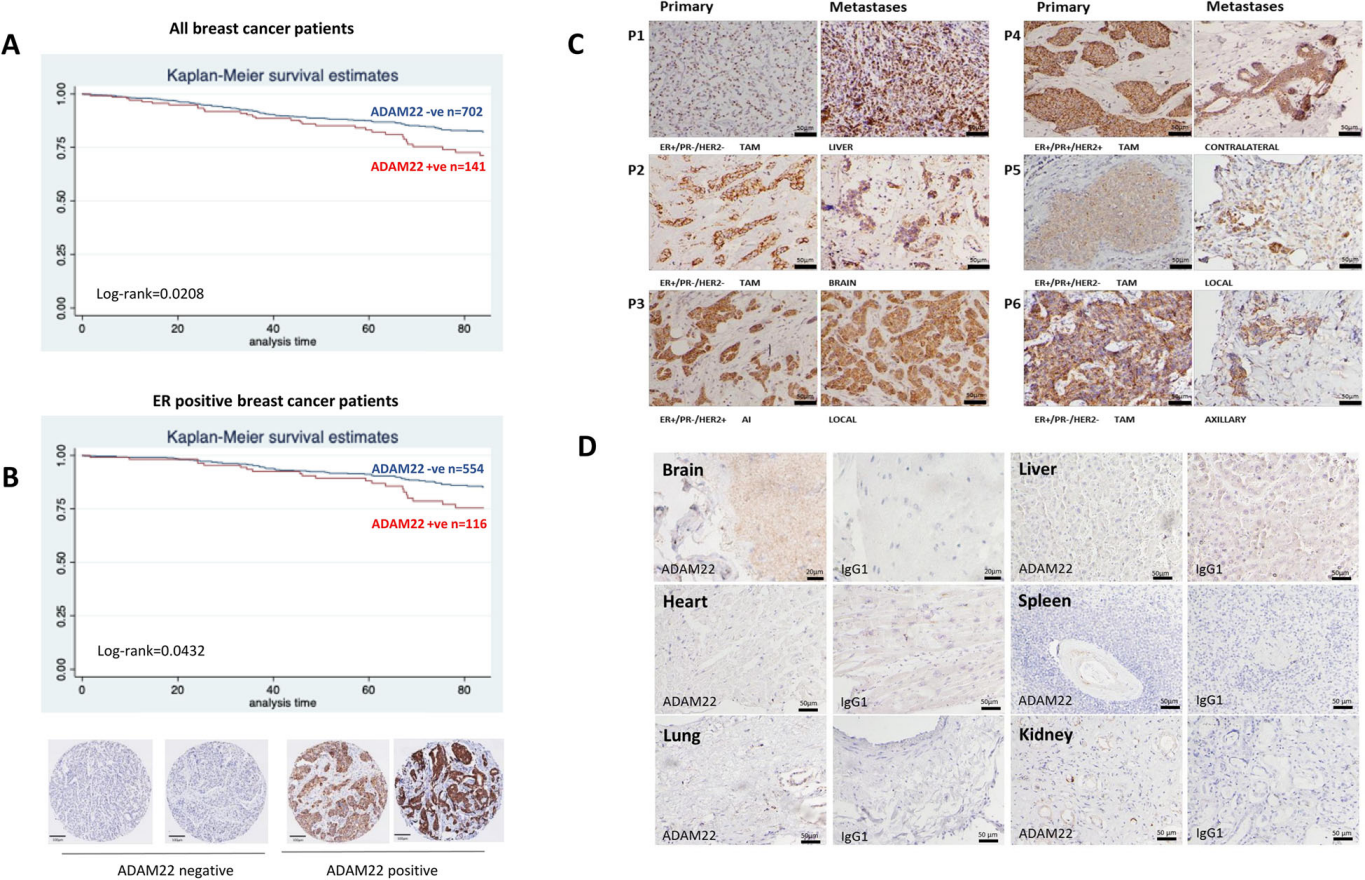
Clinically, ADAM22 expression is associated with relapse and brain metastasis and may be a suitable candidate for targeted therapy. **a** Kaplan–Meier estimates of disease-free survival according to ADAM22 in all (*n* = 843 patients) (log rank, Pr > χ^2^ = 0.0208). **b** Kaplan–Meier estimates of disease-free survival according to ADAM22 in ER-positive breast cancer patients (*n* = 670 patients) (log rank, Pr > *χ*^2^ = 0.0432). **c** Immunohistochemical ADAM22 staining in 6 ER-positive endocrine-treated primary breast tumours and matched metastatic tissue. ADAM22 expression was maintained or elevated on metastasis. Scale bar, 50 μm. **d** Immunohistochemical ADAM22 staining in normal healthy tissues. Scale bar, 20 μm for brain and 50 μm for all other organs

We have previously reported that ADAM22 protein expression in primary tumours is associated with poor disease-free survival in breast cancer patients [10]. Here we undertook ADAM22 protein target validation studies in an independent breast cancer patient cohort from a second institution (*n* = 843 patients). ADAM22 retained an association with poor disease-free survival in all breast cancer subtypes (Log-rank (*p* = 0.0208), Cox proportional hazard model (HR 1.59, 95% CI is 1.04–2.43, *p* = 0.03)) (Fig. 3a) as well as in ER-positive breast cancer patients (Log-rank (*p* = 0.0432), Cox proportional hazard model (HR 1.67, 95% CI is 1.01–2.8, *p* = 0.048)) (Fig. 3b). Cell surface expression was detected in each of the molecular subtypes with the highest rate of expression observed in ER-positive type tumours (Additional file 2 Fig. S5a). ADAM22 expression associated with clinicopathological indicators of disease progression including tumour size (*p* = 0.009) and grade (*p* = 0.02) (Additional file 2 Fig. S5b). In line with our RNAseq studies, in matched ER-positive primary tissue and metastatic tumours (liver, brain, contralateral breast, axilla and local chest wall), where ADAM22 protein was expressed in the primary tumour, it was also expressed in the metastatic tissue (Fig. 3c, Additional file 2 Fig. S5c). To test for potential on-target side effects of a putative therapeutic targeting ADAM22, we assessed expression of the protein in healthy normal organs including the liver, brain, heart, spleen, lung and kidney. No significant expression of ADAM22 was detected in these tissues (Fig. 3d).

### A peptide mimetic of LGI1 binds ADAM22 and inhibits ADAM22 driven functions in vitro and ex vivo

**Fig. 4:**
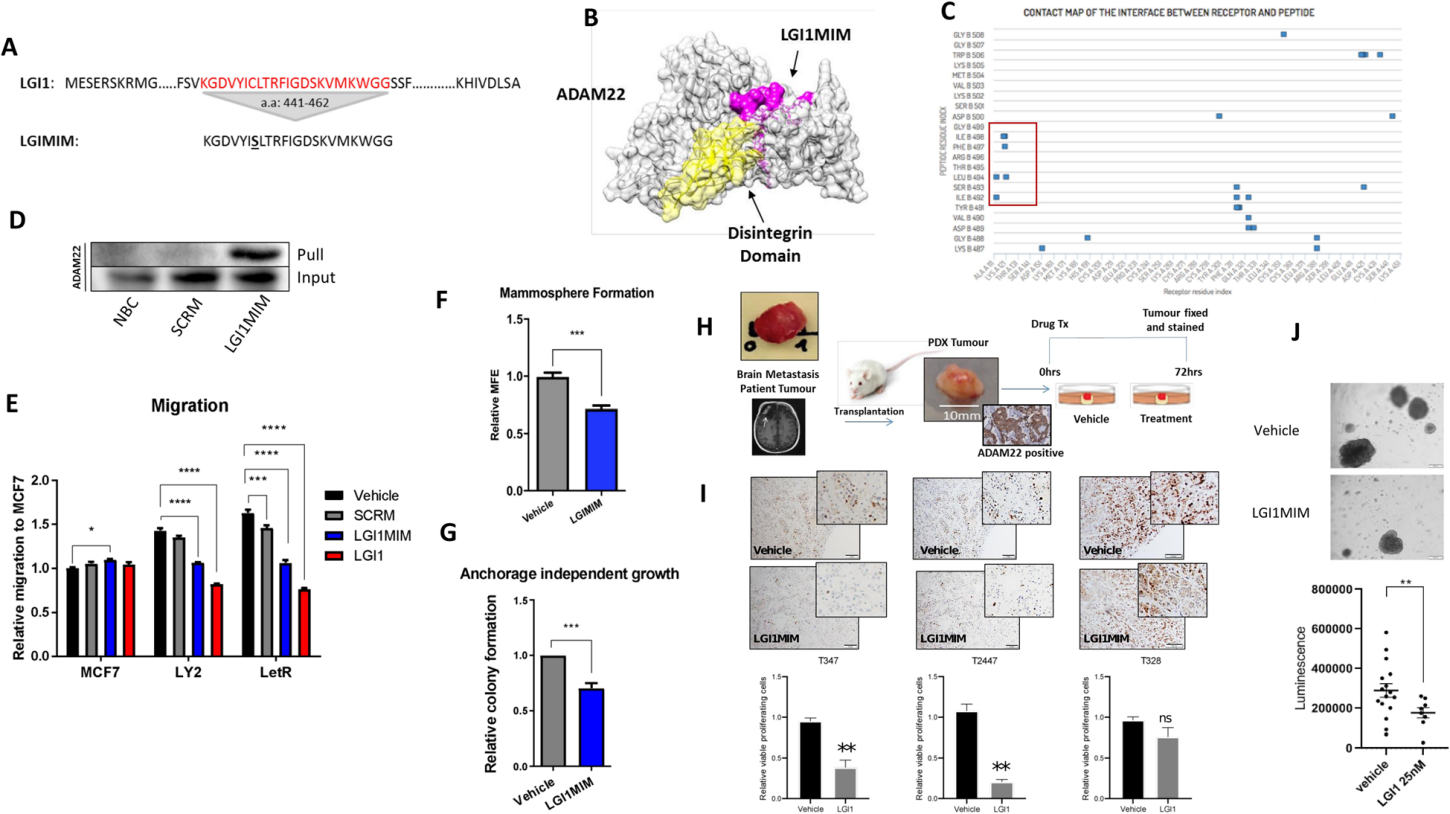
The LGI1 peptide mimetic interacts with ADAM22 and inhibits pro-metastatic potential in vitro. **a** A 22 amino acid peptide mimetic (LGI1MIM) was designed based on the predicted ADAM22 binding domain of LGI1 (amino acids 441–462). A single cysteine to serine substitution (bold and underlined) was introduced in LGI1MIM to improve solubility. **b** Predicted interaction of LGI1MIM (pink) and the disintegrin domain (yellow) of ADAM22 (grey) using CABS-dock at a threshold of < 3A. **c** Contact map showing distribution of LGI1MIM contact points within the ADAM22 protein. Contact points within the disintegrin domain are highlighted (red box). **d** Western blot confirmation of the LGI1MIM/ADAM22 interaction. A no bait control (NBC), biotinylated scrambled peptide (SCRM) and biotinylated LGI1MIM were pre-incubated with LY2 lysate before pulling associated proteins with streptavidin Dynabeads. Interacting proteins were immunoblotted with ADAM22 antibody. **e** LGI1MIM (10 nM) significantly inhibits migration of endocrine-resistant LY2 and LetR cells, similar to full length recombinant LGI1 (5 nM) compared to scrambled peptide (SCRM) or vehicle. Two-way ANOVA, *p < 0.05 ***p = 0.0002 ****p < 0.0001. **f** LGI1MIM significantly inhibits mammosphere formation. LY2 cells were plated in mammosphere-forming medium supplemented with 4-OHT (10^− 8^ M) for 5 days in the presence or absence of LGI1MIM (25 nM). Mammospheres (> 50 μm) were counted to determine the MFE. Bar graphs show relative (to untreated) MFE ± SEM from three independent experiments. Unpaired two-tailed *t*-test ***p = 0.0004. **g** LY2 cells were cultured in an anchorage independent state for 14 days in the presence or absence of LGI1MIM (25 nM). Colonies were stained with p-iodonitrotetrazolium and counted. Bar graphs show relative (to LY2) colony formation ± SEM from three independent experiments. Bar graphs show relative (to vehicle) colony formation ± SEM from three independent experiments. Unpaired two-tailed *t* test ***p = 0.0002. **h** Schematic representation of an ex vivo explant experiment testing the effect of LGI1MIM treatment on patient brain metastatic tumour. **i** Proliferation rate of the tumour cells evaluated by Ki67 immunohistochemical staining (scale bar 100 μM) and represented as relative viable proliferating cells (T347, T2447 and T328). Bar graphs show relative (to vehicle) viable cell proliferation ± SEM, *N* = 3. **j** Brain metastatic cells (T347) grown as organoids in the presence of LGI1MIM (25 nM) or vehicle for 72 h, scale bar 50 μM. LGI1MIM significantly reduced cell proliferation as measured at 7 days using a 3D cell viability assay (*p* < 0.001, *n* = 8)

LGI1 is the natural ligand for ADAM22, which binds to the receptor at its disintegrin binding domain [36], a conserved region across ADAM22 isoforms 1–5. We exploited this to develop a potential therapeutic peptide to target ADAM22-positive breast cancer cells. We designed a small 21 a.a. peptide mimetic (LGI1MIM) of the disintegrin binding domain of LGI1 with a single cysteine to serine substitution (a.a. 447) to improve solubility (Fig. 4a). The protein-peptide docking software CABS-DOCK and contact mapping modelled the LGI1MIM interaction with the disintegrin domain of ADAM22 in silico (Fig. 4b and c). LGI1MIM/ADAM22 interaction was validated in vitro using a biotin-linked LGI1MIM, which was capable of pulling ADAM22 protein from LY2 lysate (Fig. 4d). At a functional level full length LGI1 (5 nM) and LGI1MIM (10 nM) inhibited cell migration in tamoxifen-resistant LY2 cells and aromatase inhibitor-resistant LETR cells to a similar level as endocrine-sensitive MCF7 cells (Fig. 4e). LGI1MIM (25 nM) also significantly inhibited mammopshere formation and anchorage independent growth in LY2 cells (Fig. 4f and g).

To assess the efficacy of the LGI1MIM targeting ADAM22 in patient tissue, we used endocrine-resistant ER-positive, ADAM22-positive brain metastatic tumours (T347, T2447 and T328) and a triple-negative (TNBC) brain metastatic model (T2203) (Additional file 2 Fig. S6a). The brain metastatic tissue was established and expanded in a NOD/SCID mouse and tumour-specific expression of ADAM22 was confirmed in the ex vivo tissue (Fig. 4h). Treatment with LGI1MIM for 72 h had substantial anti-tumour efficacy in the ex vivo endocrine-resistant and TNBC patient models (Fig. 4i and Additional file 2 Fig. S6b, respectively) as demonstrated by a decrease in proliferating tumour cells (Ki67+) in comparison to vehicle-treated tumour tissue. In addition, 7-day treatment of ER-positive patient brain metastatic cells (T347 and T2447 cells) with LGI1MIM resulted in reduced organoid formation (*p* < 0.001, *n* = 8, *p* < 0.05, *n* = 3, respectively) (Fig. 4j and Additional file 2 Fig. S6c). Taken together, these in vitro and ex vivo studies suggest LGI1 peptide mimetic as an ADAM22-specific therapeutic with anti-tumour potential.

### LGI1MIM is a putative therapy to treat breast cancer brain metastasis

**Fig. 5:**
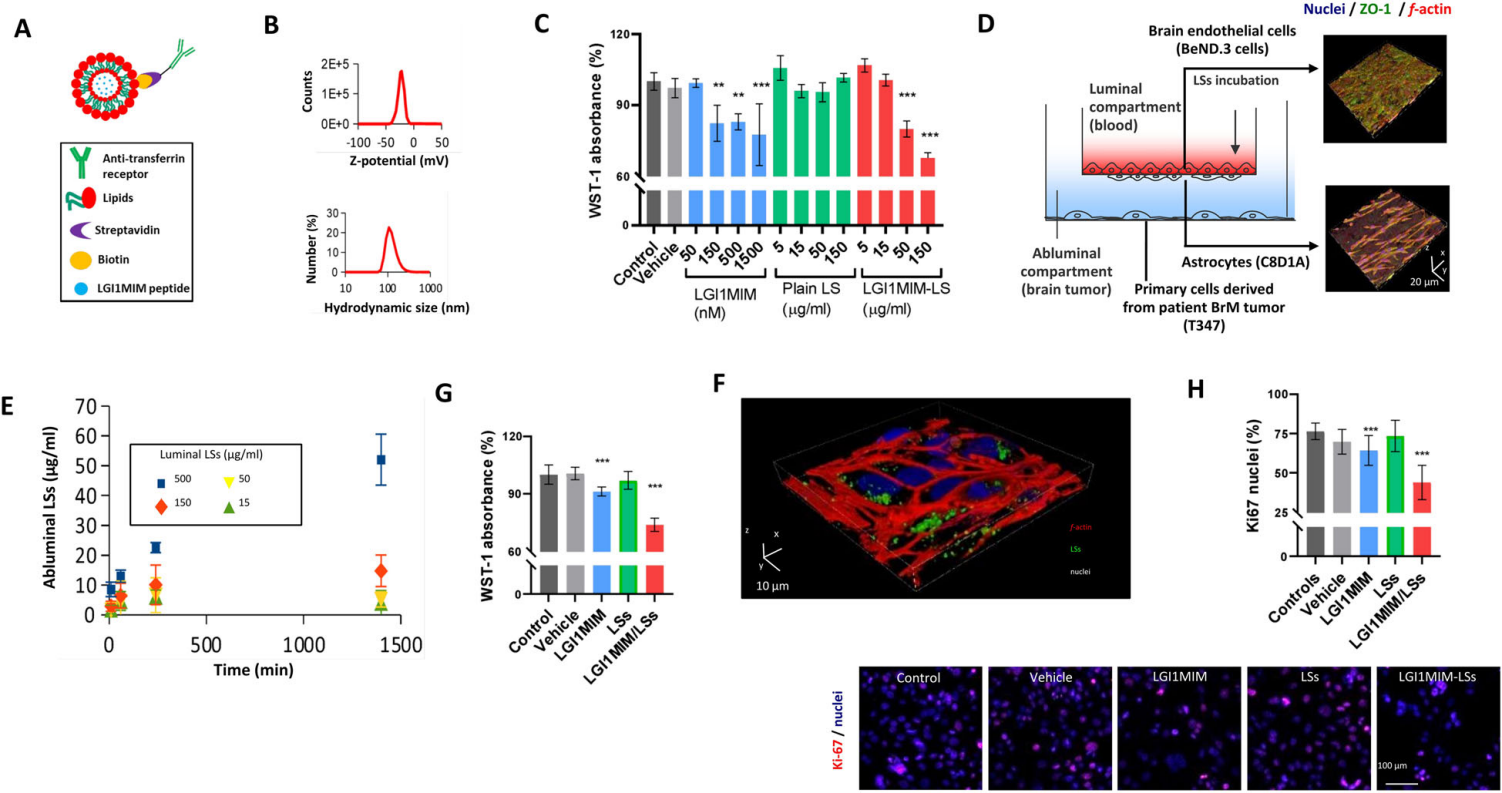
The anti-proliferative effect of LGI1MIM on metastatic tumour and the anti-cancer effect and potential of LGI1MIM-loaded liposomes (LGI1MIM/LSs) for crossing the blood–brain barrier (BBB). **a** Schematic of LGI1MIM/LSs functionalised with the antibodies against transferrin receptor. **b** Z-potential and hydrodynamic size (diameter) of the LGI1MIM/LSs. **c** WST-1 cell viability assay reveals the anti-proliferative effects of both LGI1MIM and LGI1MIM/LSs (single administrations; 72 h of treatment) on primary cells derived from brain metastatic tumour (T347). Mean ± SD *p < 0.05; **p < 0.01; ***p < 0.001, ANOVA HSD post hoc test is used. **d** Schematic of the multicellular 2D model of the BBB (left); 3D confocal imaging of the endothelial layer (top right) and of the astrocytes (bottom right); nuclei in blue, *zonula occludens-1* (ZO-1) in green and *f*-actin in red. **e** BBB crossing of DiO-stained LGI1MIM/LSs incubated in the luminal compartment at increasing concentrations (15, 50, 150 and 500 μg/ml). LGI1MIM/LSs were detected in the abluminal compartment. **f** 3D confocal laser scanning microscopy imaging of T347 cells after treatment with 500 μg/ml LGI1MIM-LSs (single administration in the luminal compartment; 72 h of incubation). **g** WST-1 cell viability assay on T347 cells in response to a single administration in the luminal compartment of vehicle, 5 μM LSs, 500 μg/ml LGI1MIM, or 500 μg/ml LGI1MIM-LSs (72 h of incubation) *p < 0.05; **p < 0.01; ***p < 0.001, ANOVA HSD post hoc test is used. **h** Ki-67 expression on T347 cells in response to the treatments reported in (k). *p < 0.05; **p < 0.01; ***p < 0.001, ANOVA HSD post hoc test is used

Drug penetration of the blood–brain barrier (BBB) represents a real clinical challenge in the development of effective treatment for brain disease. Here we have adapted a 3D biometric and biohybrid BBB model [35] to create a new patient breast cancer brain metastasis/BBB model system to test the potential of LGI1MIM as a putative therapeutic. Lipospheres (LS) were used as a carrier system for LGI1MIM (Additional file 2 Fig. S4a). The LS were functionalised with an antibody against the transferrin receptor and loaded with the LGI1MIM peptide (LGI1MIM-LS) (Fig. 5a). Transmission electron microscope imaging (Additional file 2 Fig. S4a) revealed that LGI1MIM-LSs are characterised by unilamellar morphology and homogeneous size distribution of 100 nm diameter. Dynamic light scattering analysis reported a *z*-potential of − 22.7 ± 5.1 mV, a hydrodynamic diameter of 124.6 ± 39.4 nm and a polydispersity index of 0.034 ± 0.016 (Fig. 5b). WST-1 cell proliferation assay was carried out to evaluate the biocompatibility of the control LS-based nanovectors and the effect of LGI1MIM and LGI1MIM-LSs on patient-derived brain metastatic (T347) cell viability. LGI1MIM and LGI1MIM-LS both significantly reduced T347 cell proliferation over a range of concentrations, whereas treatment with control LS had no significant effect on proliferation (Fig. 5c).

A multicellular system was used to test the functionality of LGI1MIM-LS in the context of the BBB (Fig. 5d). Brain endothelial bEnd.3 cells were seeded to cover a porous insert, developing a biological barrier highly expressing the ZO-1 tight junction marker at day 4 of culture (Fig. 5d, top right; nuclei in blue, ZO-1 in green and f-actin in red), and C8D1A astrocytes were seeded on the abluminal side of the porous insert (Fig. 5d, bottom right). The bEnd.3 cells, in combination with the C8D1A astrocytes, separated the luminal and the abluminal compartment generating a TEER of 87 ± 9 Ω cm^2^. LGI1MIM-LSs were found to successfully cross the BBB model (Fig. 5e). 3D confocal laser scanning microscopy imaging of T347 cells in the abluminal compartment demonstrated up-take of the DiO-stained LGI1MIM-LSs which crossed the BBB in vitro model (*t* = 1400 min) (Fig. 5f).

The anti-tumour efficacy of LGI1MIM-LS in the BBB model system was assessed using the WST-1 and Ki-67 proliferation assays. The luminal compartment of the system was incubated with 500 μg/ml of LGI1MIM-LSs (equivalent to 50 μg/ml in the abluminal compartment), which did not affect the integrity of the barrier (TEER values following treatment (105 ± 9 Ω·cm^2^) versus no treatment (103 ± 9 Ω·cm^2^; *p* > 0.05)). LGI1MIM and to a greater extent LGI1MIM-LS significantly reduced proliferation of breast cancer brain metastatic cells in the BBB in vitro model, with no alterations observed in the presence of control LS (Fig. 5g and h).

### LGI1MIM reduces metastatic burden in vivo and inhibits formation of brain metastases

**Fig. 6:**
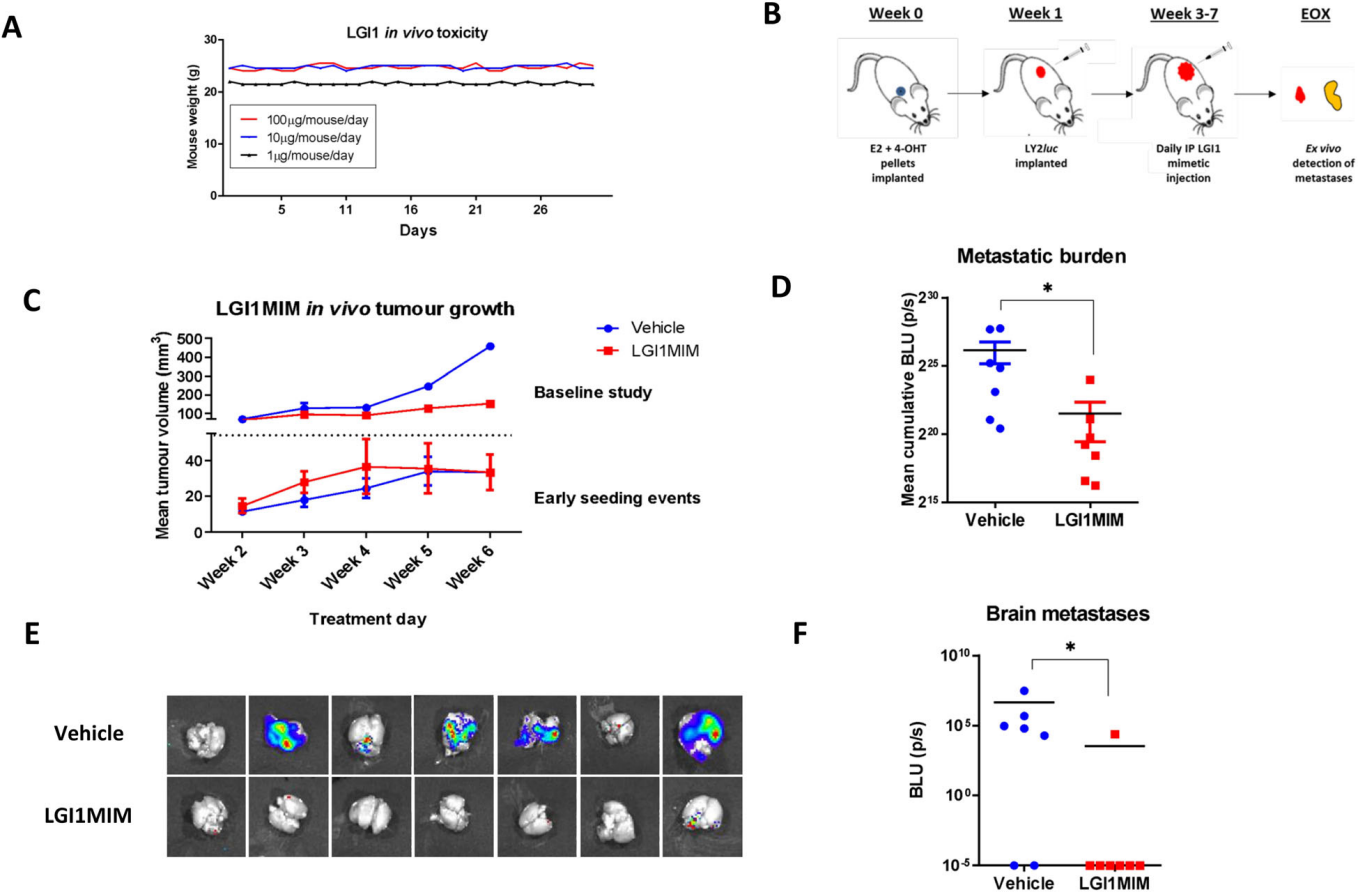
The LGI1 mimetic inhibits metastatic burden in vivo. **a** LGI1MIM displays no toxicity in mice at low-, mid- and high-dose concentrations. NOD/SCID mice were administered a daily regimen of LGI1MIM by IP injection at three doses: 1 μg/mouse/day (*n* = 2), 10 μg/mouse/day (*n* = 2) and 100 μg/mouse/day (*n* = 2). Mouse weights were noted daily as a measure of LGI1MIM tolerability. **b** Schematic of LGI1MIM in vivo study to examine early metastatic seeding events. **c** Mean tumour volume in baseline study (top) determines baseline LGI1MIM tumour inhibition and the early seeding study (bottom) determines LGI1MIM effect on initial metastatic events (LGI1MIM = red; vehicle = blue). Either 1 × 10^6^ (baseline study) or 8 × 10^5^ (early seeding study) luciferase tagged LY2 cells were implanted into the mammary fat pad of NOD/SCID mice. Mice were treated with either vehicle (*n* = 2 mice baseline study; *n* = 7 mice early seeding study) or LGI1MIM at (100 μg/mouse/per day) (*n* = 2 control study; *n* = 7 metastatic study) for 6 weeks. LGI1MIM induced a substantial decrease in local tumour volume in the baseline study (mean 450 mm^3^ versus 150 mm^3^, calliper measurement), whereas no alteration was detected in the early metastatic seeding model. **d** Cumulative metastatic burden in vehicle- and LGI1MIM-treated mice. LGI1MIM significantly reduces metastatic burden (as measured by BLU log^2^ p/s). Two tailed Mann–Whitney test* *p = 0.0111.* **e** The ex vivo luciferase activity from brain metastases in vehicle-treated (top) and LGI1MIM-treated (bottom) mice. **f** Brain metastatic burden, BLU log^2^ p/s, was significantly reduced in LGI1MIM treated mice (red; *n* = 7 mice) versus vehicle control (blue; *n* = 7 mice). Two-tailed Mann–Whitney test* *p = 0.0373*

Having established the ability of LGI1MIM to cross the BBB and reduce tumour cell proliferation, the efficacy of LGI1MIM as a therapeutic was further evaluated in an orthotopic xenograft model of endocrine resistance. LGI1MIM tolerance was first investigated in vivo, with no observable loss of weight detected at any of the concentrations tested (Fig. 6a).

In an endocrine-resistant xenograft model, LY2 *luc* cells were implanted into the mammary fat pad of NOD/SCID mice and the tumours allowed to develop. At week 5, tumours were resected and animals were treated with either LGI1MIM or vehicle. LGI1MIM inhibited local disease recurrence post resection and prevented formation of distant metastatic disease of the brain and other organs including the lung, liver and bone (Additional file 2 Fig. S7a).

At a clinical level, early intervention to prevent the development of overt brain metastasis and corresponding neuronal complications is optimal. A second study was undertaken to determine the efficacy of LGI1MIM to inhibit early metastatic events (Fig. 6b). To establish baseline tumour inhibition, LY2 *luc* cells were implanted into the mammary fat pad of NOD/SCID mice (Fig. 6c). Tumours were allowed to develop and animals were then treated with LGI1MIM or vehicle for 6 weeks. LGI1 substantially reduced local tumour growth (mean 450 mm^3^ versus 150 mm^3^) (Fig. 6c), in a similar manner to that observed with the LY2-ADAM22 KO cells (Fig. 2a). To evaluate early metastatic events, we implanted a reduced number of LY2 *luc* cells into the mammary fat pad of the mice to maintain low primary tumour volume. Tumours were allowed to establish until palpable and animals were then treated with either LGI1MIM or vehicle (Fig. 6c). Mammary fat pad tumours were small (mean 32 mm^3^) and no difference in tumour volume was observed between the treatment groups (Fig. 6c, Additional file 2 Fig. S7b). Distant metastatic disease formation however was significantly reduced in the LGI1MIM treatment arm as determined by ex vivo IVIS imaging (Fig. 6d and Additional file 2 Fig. S7c). Moreover, targeting ADAM22 with LGI1 induced consistent reductions in micro-metastatic disease burden in the brain (Fig. 6e and f).

## Discussion

Individual site-specific metastatic studies describe alterations in transcriptomic signalling, including ER expression/tyrosine kinase signalling in the brain [3], CDK/Rb/E2F and FGFR in the bone [20] and ECM interactions in the liver [2, 35]. Here collective analysis of differentially regulated genes in matched primary tumour and corresponding bone, brain and liver metastasis from breast cancer patients revealed [2, 37] that ECM and extracellular structure organisation were shared processes with the greatest enhancement on metastasis. The ADAM proteins belong to the reprolysin family and are thought to play a key role in regulating cell–cell communication [8]. We have previously described a role for ADAM22 in endocrine-resistant breast cancer, and in this study, we have further demonstrated a gain in ADAM22 expression in ER-positive brain metastases. ADAM22 can enhance cell migration and inhibit differentiation in resistant model systems and is an independent predictor of poor disease-free survival in breast cancer patients [10]. Here, ADAM22 knock-out and knock-in studies in xenograft models of endocrine resistance define a new role for ADAM22 in disease progression and metastasis.

The contribution of ADAMs to disease progression has largely been attributed to their proteolytic activity mediated through a functional metalloproteinase domain [14, 38]. ADAM22 however lacks an active metalloproteinase site and has no proteolytic activity [14]. Though there has been little study to date on the non-catalytic function of ADAM proteins in cancer, a role for ADAM22 in cell adhesion and spreading in conjunction with the 14-3-3 family of signalling proteins has been described [39–41]. Here an RPPA approach was used to assess ADAM22-dependent signalling in endocrine-resistant breast cancer. Cell-to-cell communication and growth factor pathway activation, including HER2 signalling, VEGF and IGFR, were observed as key ADAM22-associated networks. The significance of an ADAM17-EGFR axis in mammary gland development and cancer has previously been described [42]. ADAM22 may use similar signalling mechanisms to drive metastatic processes including stem-like activity and anchorage independent growth observed in this study, potentially through its cysteine-rich/EGF-like domains.

We assessed the potential of ADAM22 as a therapeutic target. Robust cell surface ADAM22 protein was found in approximately 17% of primary breast cancer tissues across all of the molecular sub-types. Elevation of ADAM22 transcript was observed in metastatic tumours in comparison to matched primary tissue, particularly in breast to brain metastasis, as evidenced in both transcript and protein. For any potential drug target, it is necessary to assess the on-target side effects [43]. Here potential ADAM22 on-target side effect was considered low, as insignificant ADAM22 protein expression was observed in normal healthy organs in comparison to primary and metastatic breast cancer tissue. However more in-depth toxicity studies will need to be undertaken to establish the full safety profile of ADAM22 targeted therapies.

ADAM22 can interact with extracellular proteins to alter cell processes. LGI1 has been shown to act as a specific extra-cellular ligand for ADAM22 to regulate synaptic transmission through stabilisation of the AMPA/stargazin complex [36]. The LGI1-ADAM22 ligand/receptor complex has been suggested as a potential therapeutic target to treat synaptic disorders in the nervous system [13]. In cancer, a tumour suppressor role for LGI1 has been described in glioblastoma and neuroblastoma, and it has been shown that LGI1 may play a role in impairing proliferation and survival in HeLa cells [44–46]. We have previously reported LGI1-induced inhibition of cell migration in endocrine-resistant breast cancer cells [10]. Furthermore, in this study, analysis of metastatic dominant ECM genes in a cohort of matched primary and metastatic tumours revealed ADAM22 as a top member of the druggable genome. These data raise the possibility of LGI1/ADAM22 as a therapeutic complex to treat advanced breast cancer.

There has been an increased interest in use of therapeutic peptides as they have demonstrated to be highly selective and efficacious, with low cytotoxicity [47]. Of interest, therapeutic peptides have shown better efficacy in comparison to antibody-based therapies in penetrating blood–brain barrier and for treatment of brain cancers [48, 49]. To exploit LGI1 as a therapeutic, we designed a small peptide mimetic against the ligand binding pocket of LGI1 in the disintegrin binding domain of ADAM22. Successful binding was confirmed in silico and in vitro. At a functional level, the peptide had similar effect as full length LGI1, halting cell migration of aggressive endocrine-resistant cells to a comparable level of that observed in endocrine-sensitive MCF7 cells. To further asses the efficacy of LGI in targeting ADAM22 in breast cancer patient samples, an ADAM22-positive brain metastatic patient model was used [3]. Treatment of this patient ex vivo model with LGI1MIM demonstrated an anti-tumourigenic efficacy as evidenced by reductions in the proliferation marker Ki67.

Treatment of breast cancer brain metastasis is a significant challenge, in particular with regard to ensuring successful drug penetration of the BBB [50]. Given the elevated levels of ADAM22 in breast cancer brain metastatic tumours and the efficacy of LGI1MIM in inhibiting early metastatic events, we evaluated the potential of LGI1/ADAM22 complex as a new therapeutic strategy in breast cancer-BBB model systems. Model systems to test new treatments are challenging, and none is without limitation; here we test LGI1 in a patient-derived system. We adapted a 3D biometric and biohybrid system [35] to create a multi-cellular breast cancer BBB model, encompassing endothelial cells, astrocytes and patient-derived brain metastatic tumour cells. This model recapitulates the dynamics of the BBB in vivo and has the potential for assessing the efficacy of new brain cancer therapeutics beyond this study. In this model system LGI1MIM and in particular LGI1MIM-LS were found to successfully cross the BBB and inhibit tumour cell proliferation.

The ability of LGI1MIM to target ADAM22 either in the primary or metastatic site to inhibit the development of disease progression was demonstrated in vivo*.* Given that early intervention is crucial in combating progression to metastasis [51], further clinical utility of the drug was assessed in the micro-metastatic setting. The LGI1MIM prevented early progression of breast cancer brain metastasis in our endocrine-resistant breast cancer xenograft model. Though these in vivo studies provide considerable evidence of LGI1MIM as a putative therapeutic to treat the development of brain metastasis, further studies investigating drug metabolism as well as on-target side effects in normal brain tissue are required.

## Conclusion

Recurrent elevations in ECM organisation networks are observed on breast cancer metastasis. Data presented in this study suggest that targeting the ECM signalling protein ADAM22 may represent a new therapeutic strategy to treat breast cancer brain metastasis.

## Supplementary information

**Additional file 1:** Supplemental Methods.

**Additional file 2: Figure S1.** Confirmation of LY2 ADAM22 CRISPR knockout cell line. (a) RFP expression confirming CRISPR /Cas9 mediated double stranded break and insertion of an RFP tagged HDR plasmid (b) FACS gating strategy based on high RFP expression to single cell sort CRISPR/Cas9 ADAM22 KO cells (right) from LY2 WT cells (left) (c) Confirmation of ADAM22 gene silencing in an LY2 ADAM22 KO clone (Clone H (C-H)) by PCR and Western blot analysis (*n* = 4). *** *p* < 0.0005 (d) Genotyping of Clone H using primers which flank the ADAM22 CRISPR sgRNA sites 1 and 3 (left) and sgRNA 2 (right). Clone H contains a homozygous deletion at cut site 1 & 3 and a heterozygous HDR insertion at cut site 2. (e) Sanger sequencing of the homozygous 58 bp deletion in Clone H (red) flanked by the sgRNA 1 and 3 cut sites (lilac) in LY2 WT cells. **Figure S2.** Confirmation of LY2 lentiviral ADAM22 knock in (KI) cell line. (a) GFP expression confirming successful transduction of LY2 cells with lentiviral ADAM22 particles. (b) FACS gating strategy based on high GFP expression to purify LY2 ADAM22 KI cells (right) from LY2 WT cells (left). (c) Confirmation of ADAM22 gene overexpression in the LY2 lentiADAM22 KI cell line. *p < 0.05. (d) Western blot confirmation of ADAM22 overexpression in LY2 lentiADAM22 cells versus LY2 WT cells. **Figure S3.** ADAM22 KO, KI and WT cells. (a) ADAM22 protein expression in each biological replicate used for the RPPA study. (b) IVIS imaging of luciferase activity in LY2 luc, Clone H luc (ADAM22 KO) and lentiA22 luc (ADAM22 KI) cells in vitro after treatment with 15 μg/ml of luciferin. (c) Luciferase activity was comparable across each cell line with respect to cell number. (d) Tumour weight was significantly reduced in LY2 ADAM22 KO tumours versus LY2 WT. Tumour weight ± SEM, Unpaired Mann Whitney two tailed t test *p < 0.05. **Figure S4.** Quantification of LGIMIM peptide in LSs. (a) Transmission electron microscopy (TEM) imaging of LGI1MIM-LSs and SDS-page stained with Coomassie Blue of LGI1MIM (6.0, 2.0, 0.7 and 0.2 μg), empty LSs (5 mg/ml) (b) Calibration curve reporting the band intensities (pixel values) for different amounts of LGI1MIM. **Figure S5.** ADAM22 clinical data. (a) ADAM22 expression was examined in a cohort of 843 breast cancer patients and the percentage of ADAM22-positive is stated in each of the breast cancer subtypes as well as in patients with recurrent disease. (b) Associations of ADAM22 in TMA of breast cancer patients with clinicopathologic variables using chi-square test. (C) Clinical molecular status, treatment data and H-score from primary and metastatic patient tumours. **Figure S6.** A peptide mimetic of LGI1 inhibits ADAM22 driven functions in vitro and ex vivo (a) Clinical molecular status of patient tumours. (b) Proliferation rate of the tumour cells from a triple negative tumour (T2203) in an ex vivo explant evaluated by Ki67 immunohistochemical staining (scale bar100μM) and represented as relative viable proliferating cells. (b) Brain metastatic cells from an ER positive patient tumour (T2447) grown as organoids in the presence of LGI1MIM (25 nM) or vehicle for 72 h, scale bar 50 μM. LGI1MIM significantly reduced cell proliferation as measured at 7 days using a 3D cell viability assay (*P* = 0.0373, *n* = 3). **Figure S7.** LGI1MIM inhibits disease progression in endocrine resistant xenograft models. (a) Local and distant metastatic disease were assessed in a resection endocrine resistant xenograft model. 1X10^6^ luciferase tagged LY2 cells were injected into the left inguinal mammary fat pad of NOD/SCID mice Tumours were allowed to form and at week 5, primary tumours were surgically removed. Mice were treated either with vehicle (n = 3 mice) (top) or LGI1MIM (100 μg/mouse/day) (*n* = 2 mice) (bottom) for 6 weeks. Tumour growth was monitored using calliper measurement. Mice were culled at the end of the experimental period (15 weeks) or once the tumour reached 500 mm3. Local (in vivo) and distant organ specific (ex vivo) recurrences were examined using an IVIS imaging system. (b) Weekly IVIS readings of local tumour burden in early seeding study. 800X10^5^ luciferase tagged LY2 cells were implanted into the mammary fat pad of NOD SCID mice. Mice were treated with either vehicle (*n* = 7 mice metastatic study) or LGI1MIM at (100 μg/mouse/per day) (n = 7 metastatic study). No differences in tumour cells (mean BLU (p/s) scores) was detected in vehicle versus LGI1MIM treatment groups. (c) Representative image of ex vivo luciferase activity from Lung, bone, liver and brain metastases in vehicle treated (left) and LGI1MIM treated (right) mice. No significant differences in metastatic burden were observed in liver and lung, Bone and brain showed reduced metastatic burden in LGI1MIM treated mice compared to vehicle treated mice, BLU log2 p/s, (n = 7 mice) Two tailed Mann Whitney test** *p* = 0.007, bone and *p = 0.0373 brain.

**Additional file 3: Table S1.** Differential gene expression (log2FC +/− 1.5 adjusted pval < 0.05) metastases vs primary sets used as input for Gene Ontology Enrichment Testing with matrisomeDB annotation.

**Additional file 4: Table S2.** Differentially expressed protein coding genes (log2FC +/− 1.5) in ER+ primary breast and matched brain metastatic tumours (*N* = 9 patients).

**Additional file 5: Table S3.** ER+ subtype patient specific fold-changes and expression gains in drug targetable matrisome associated genes (N = 9 patients).

## Data Availability

All data will be made available and uploaded to the relevant publicly accessible databases.
