# Correction: ADAM22/LGI1 complex as a new actionable target for breast cancer brain metastasis

**DOI:** 10.1186/s12916-025-04578-x

**Published:** 2025-12-13

**Authors:** Sara Charmsaz, Ben Doherty, Sinéad Cocchiglia, Damir Varešlija, Attilio Marino, Nicola Cosgrove, Ricardo Marques, Nolan Priedigkeit, Siobhan Purcell, Fiona Bane, Jarlath Bolger, Christopher Byrne, Philip J. O’Halloran, Francesca Brett, Katherine Sheehan, Kieran Brennan, Ann M. Hopkins, Stephen Keelan, Petra Jagust, Stephen Madden, Chiara Martinelli, Matteo Battaglini, Steffi Oesterreich, Adrian V. Lee, Gianni Ciofani, Arnold D. K. Hill, Leonie S. Young

**Affiliations:** 1 Endocrine Oncology Research Group, Department of Surgery, Royal College of Surgeons in Ireland, Dublin 2, Ireland; 2 Smart Bio-Interfaces, Istituto Italiano di Tecnologia, Scuola Superiore Sant’Anna, Pontedera, Italy; 3 Women’s Cancer Research Centre, Magee-Women’s Research Institute, University of Pittsburgh Cancer Institute, University of Pittsburgh, Pittsburgh, PA USA; 4 Department of Neurosurgery, National Neurosurgical Centre, Beaumont Hospital, Dublin, Ireland; 5 Department of Neuropathology, Beaumont Hospital, Dublin, Ireland; 6 Department of Pathology, Royal College of Surgeons in Ireland, Dublin, Ireland; 7 Department of Surgery, Royal College of Surgeons in Ireland, Dublin, Ireland; 8 Data Science Centre, Royal College of Surgeons in Ireland, Dublin, Ireland; 9 The Biorobotics Institute, Scuola Superiore Sant’Anna, Pontedera, Italy; 10 Department of Surgery, Beaumont Hospital, Dublin, Ireland

**Correction: BMC Med 18, 349 (2020)**

**https://doi.org/10.1186/s12916-020-01806-4**

Original article published: 19 November 2020. Volume 18, article number 349, (2020)

The authors of the original article [1], wish to clarify an error affecting Fig. 4I.

The image derived from Fig. 4I, (the immunohistochemistry images showing Ki67 staining in the T347 tumor), was erroneous due to an upload error during manuscript revisions.

The authors reviewed all experimental data and image files and identified the correct images. The graph of the quantification in the original figure remains the same, as that analysis was done on the correct images. The corrected Fig. 4I presents the accurate Ki67 immunohistochemistry for the T347 tumor.

**Fig. 4:**
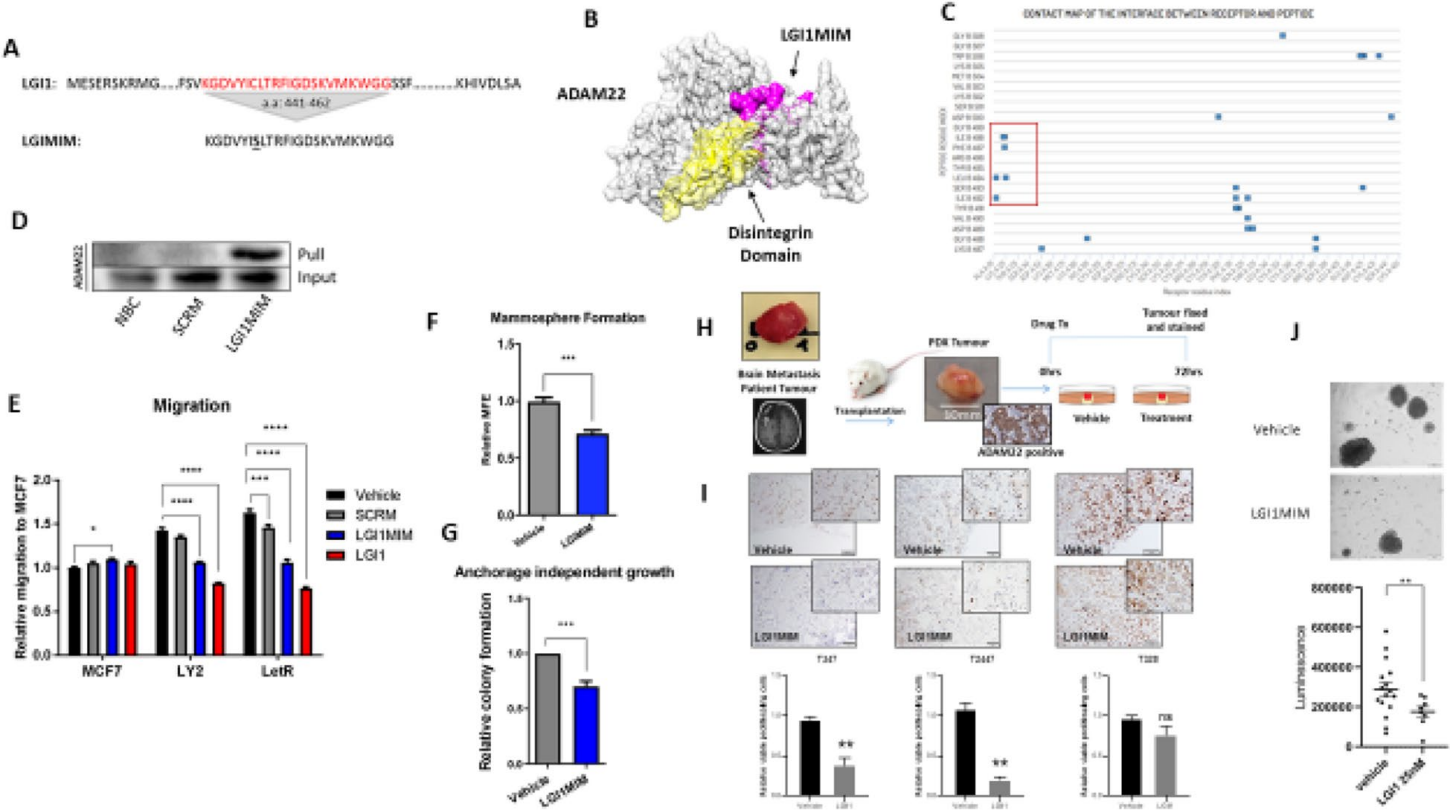
The LGI1 peptide mimetic interacts with ADAM22 and inhibits pro-metastatic potential in vitro. **a** A 22 amino acid peptide mimetic (LGI1MIM) was designed based on the predicted ADAM22 binding domain of LGI1 (amino acids 441–462). A single cysteine to serine substitution (bold and underlined) was introduced in LGI1MIM to improve solubility. **b** Predicted interaction of LGI1MIM (pink) and the disintegrin domain (yellow) of ADAM22 (grey) using CABS-dock at a threshold of < 3A. **c** Contact map showing distribution of LGI1MIM contact points within the ADAM22 protein. Contact points within the disintegrin domain are highlighted (red box). **d** Western blot confirmation of the LGI1MIM/ADAM22 interaction. A no bait control (NBC), biotinylated scrambled peptide (SCRM) and biotinylated LGI1MIM were pre-incubated with LY2 lysate before pulling associated proteins with streptavidin Dynabeads. Interacting proteins were immunoblotted with ADAM22 antibody. **e** LGI1MIM (10 nM) significantly inhibits migration of endocrine-resistant LY2 and LetR cells, similar to full length recombinant LGI1 (5 nM) compared to scrambled peptide (SCRM) or vehicle. Two-way ANOVA, *p < *0.05* ***p = *0.0002* ****p < *0.0001*. **f** LGI1MIM significantly inhibits mammosphere formation. LY2 cells were plated in mammosphere-forming medium supplemented with 4-OHT (10^− 8^ M) for 5 days in the presence or absence of LGI1MIM (25 nM). Mammospheres (> 50 μm) were counted to determine the MFE. Bar graphs show relative (to untreated) MFE ± SEM from three independent experiments. Unpaired two-tailed t-test ***p = *0.0004*. **g** LY2 cells were cultured in an anchorage independent state for 14 days in the presence or absence of LGI1MIM (25 nM). Colonies were stained with p-iodonitrotetrazolium and counted. Bar graphs show relative (to LY2) colony formation ± SEM from three independent experiments. Bar graphs show relative (to vehicle) colony formation ± SEM from three independent experiments. Unpaired two-tailed t test ***p = *0.0002*. **h** Schematic representation of an ex vivo explant experiment testing the effect of LGI1MIM treatment on patient brain metastatic tumour. **i** Proliferation rate of the tumour cells evaluated by Ki67 immunohistochemical staining (scale bar 100 μM) and represented as relative viable proliferating cells (T347, T2447 and T328). Bar graphs show relative (to vehicle) viable cell proliferation ± SEM, *N* = 3. **j** Brain metastatic cells (T347) grown as organoids in the presence of LGI1MIM (25 nM) or vehicle for 72 h, scale bar 50 μM. LGI1MIM significantly reduced cell proliferation as measured at 7 days using a 3D cell viability assay (*p* < 0.001, *n* = 8)
