# Supplementary material for: ADAM22/LGI1 complex as a new actionable target for breast cancer brain metastasis
Source: BMC Med. 2020 Nov 19;18:349. doi: 10.1186/s12916-020-01806-4 (PMC7677775; doi:10.1186/s12916-020-01806-4)
Supplement: Supplementary file 1 — Additional file 1: Supplemental Methods. [file 12916_2020_1806_MOESM1_ESM.docx]

**Supplemental Methods**

**ADAM22 lentiviral transduction and CRISPR/Cas9 models**

LY2 ADAM22 CRISPR/Cas9 transfected cells were selected under puromycin dihydrochloride (8 μg/ml) (sc-108071, Santa Cruz) (Supplementary Fig. 1a) and single cell sorted by fluorescently activated cell sorting (FACS) (Supplementary Fig. 1b). The LY2-ADAM22 selected CRISPR/Cas9 clonal gDNA were genotyped by PCR, western blot and sequenced by Sanger sequencing to confirm a clone with bi-allelic ADAM22 KO (Supplementary Fig. 1c, 1d). The LY2 ADAM22 KO was confirmed by quantitative polymerase chain reaction (qPCR) (Supplementary Fig. 1e). Cells were maintained in LY2 media supplemented with 4 μg/ml of puromycin.

The ADAM22 overexpressing cell line was stablished by transfecting the LY2 cells with lentiviral particles, containing recombinant ADAM22 variant 1 with a GFP c-terminal fusion tag (RC219272L2V, OriGene / AMSBIO) (Supplementary Fig. 2a). LY2 ADAM22 KI cells were selected for high ADAM22 expressing cells by FACS (Supplementary Fig. 2b). The level of ADAM22 overexpression was confirmed both at transcript and protein level (Supplementary Fig. 2c, 2d).

**Reverse phase proteomic study (RPPA)**

RPPA analysis was carried out on protein lysates from LY2 and LY2 ADAM22 KO cells which were diluted to a final concentration of 1 mg/ml. The level of ADAM22 was evaluated between each biological replicate of LY2 ADAM22 KO and the LY2 cells (Supplementary Fig. 3a). Lysates were then spotted on nitrocellulose-coated FAST slides (Whatman, Schleicher & Schuell BioScience) by a Genetix QArray2 spotter. Samples were probed by the CSA amplification approach and visualised by DAB colorimetric reaction. Slides were then scanned on a flatbed scanner, spots were identified and their density was quantified by MicroVigene. All the data points were normalised for protein loading and transformed to a linear value before final analysis. The RPPA dataset was first formatted according to the RPPApipe online protocol to include gene symbols and modifications and saved as a text document. After uploading to the RPPApipe server, biological replicates were assigned to defined sample groups (e.g LY2 vs Clone H). These sample groups were then mean-centred and processed using a linear model (LIMMA) statistical method to determine differentially expressed genes between the two subgroups, which were displayed as fold differences with associated p-values. In order to examine ADAM22 associated pathways, the list of differentially expressed proteins was uploaded in ClueGO plugin from Cytoscape (v.3.4.0) to generate Kegg pathways associated with ADAM22 protein expression. In another approach upregulated proteins in the parental LY2 cell line were assigned unique UniProt accession numbers and uploaded to the DAVID functional annotation tool.

**Peptide synthesis**

ADAM22/LGI1 mimetic *in silico* interaction ADAM22 and LGI1 mimetic predicted 3D structures were obtained from the I-TASSER server ^11^. Molecular docking studies were then carried out using the CABS-dock server, using a high quality prediction of < 3 Ångströms ^12^.

***In vitro* luciferase activity**

Cells were treated with XenoLight D-Luciferin 15 µg/ml of luciferin (122799, PerkinElmer) and luciferase activity was detected using the IVIS system (Xenogen, PerkinElmer) as per manufacturer instruction (Supplementary Fig. 3b, 3c).

**In vivo studies**

Animals: Female NOD/SCID mice 21-29 days old were used for all experiments. Animals acclimatised for 2 week prior to commencement of experimental procedures. Humane endpoint was either maximum tumour volume or experimental period of 15 weeks. Animals were housed with a maximum of 5 per cage. Enrichment was provided in the form of plastic housing and nesting material. In total 37 animals were used throughout the study.

ADAM22 Study: ADAM22 contribution to tumour progression was determined using an *in vivo* model of endocrine resistant breast cancer. 1X10^6^ luciferase tagged LY2, LY2 ADAM22 KO and LY2 ADAM22 KI cells were injected into the left inguinal mammary fat pad of NOD/SCID mice (n = 8, n = 7 and n= 7, respectively), supplemented with slow release 17β-estradiol (1 mg/pellet) and tamoxifen pellets (5 mg/pellet; Innovative Research of America). Animals were assigned to experimental groups randomly and were mixed within cages. Tumour growth was monitored by calliper measurement and IVIS imaging on alternate days (Supplementary Fig. 3d). At the experimental end point (tumour volume, 150 mm^3^) primary tumour were measured (Supplementary Fig. 3e) and imaged *ex vivo* by IVIS. Researchers conducted all measurements blinded. Statistical analysis was carried out using Graphpad Prism as described in the Results section.

LGI1MIM Toxicity study**:**

LGI1MIM toxicity was tested in NOD/SCID mice. The mice were injected with either 100, 10 or 1ug/kg/day for 30 days (n=6, 2 mice per treatment concentration). Mouse health (behaviour, gate and weight) was monitored daily.

LGI1MIM resection study:

To examine the effect of LGI1MIM on metastasis a study was conducted in which 1X10^6^ luciferase tagged LY2 cells were injected into the left inguinal mammary fat pad of NOD/SCID mice supplemented with 17β-estradiol (1 mg/pellet) and tamoxifen (5 mg/pellet) pellets. The xenografts were allowed to form tumours and at week 5, primary tumours were surgically removed. Mice were randomised into two daily treatment arms: vehicle (n=3 mice) or LGI1MIM (100μg/mouse/day) (n=2 mice) for 6 weeks (Supplementary Fig. 5a). Tumour growth was monitored using calliper measurement. Mice were culled at the end of the experimental period (15 weeks) or once the tumour reached 500 mm^3^. Local and distant recurrences were examined using an IVIS imaging system *in vivo* and *ex vivo,* respectively (Supplementary Fig. 5a). Researchers conducted all measurements and data analysis.

LGI1MIM baseline xenograft:

A control study was set-up to confirm LGI1MIM’s inhibitory effect on tumour volume. 1X10^6^ luciferase tagged LY2 cells were injected into the left inguinal mammary fat pad of NOD/SCID mice supplemented with 17β-estradiol (1 mg/pellet) and tamoxifen (5 mg/pellet) pellets. Tumours were allowed to develop to ~70mm^3^. Mice were randomised into two treatment arms and received daily treatment of either vehicle (n=2 mice) or LGI1MIM (100 μg/mouse/day) (n=2 mice) for 6 weeks. Tumour growth was monitored using calliper measurement. Mice were culled at the end of the treatment period or once the tumour reached 500 mm^3^. Researchers conducted all measurements and data analysis.

LGI1MIM early metastatic study:

To assess the effect of LGI1MIM on initial metastatic events an early seeding *in vivo* study was set up. In this study 8x10^5^ luciferase tagged LY2 cells were injected into the left inguinal mammary fat pad of NOD/SCID mice supplemented with 17β-estradiol (1 mg/pellet) and tamoxifen (5 mg/pellet) pellets. Xenografts were monitored for tumour formation by IVIS. Once the tumour was palpable mice were randomised to receive daily treatment of either vehicle (n=7 mice) or LGI1MIM (100 μg/mouse/day) (n=7 mice) for 6 weeks. Treatment groups were mixed within a cage. Tumour growth was monitored using calliper measurement and IVIS imaging (Supplementary Fig. 5b). Mice were culled at the end of the treatment period. Organ specific metastasis (bone, brain, liver and lung) were assessed *ex vivo* by IVIS (Supplementary Fig. 5c). Researchers conducted all measurements and data analysis.

**Biomimetic LGI1MIM-loaded liposome (LGI1MIM-LS) preparation, functionalization and staining**

LGI1MIM-LSs were obtained using the thin-film evaporation method. 2.5 mg of brain lipids (Avanti Polar Lipids, Inc.) and 5 mg of a biotin-conjugated 1,2-distearoyl-sn-glycero-3-phosphoethanolamine-N- (polyethylenglycol)-5000 (biotin-DSPE-PEG5000) were dissolved in 1 ml of chloroform and transferred for solvent evaporation. The dried lipids were then re-hydrated with 1 ml of ddH_2_O in the presence of 185 µM LGI1MIM-LSs.

Non-loaded LSs were used as control. Obtained dispersion was extruded with 11 passages (Avanti® Mini Extruder) through a polycarbonate membrane of 0.1 μm pore size. LSs were purified by dialysis from the non-encapsulated LGI1MIM (overnight under stirring with ddH_2_O as eluent; molecular weight cut off 300 KDa; Spectrum laboratories, Inc.). Finally, LGI1MIM-LSs were functionalized with a streptavidine-conjugated anti-transferrin receptor antibody (anti-TfR; 25 µg/ml), and purified as previously described ^13^. For the evaluation of blood-brain barrier (BBB) crossing, 5 mg/ml of LSs were stained with Vybrant™ DiO labeling solution (1:25 dilution) and purified overnight as described above.

**LGI1MIM-LS imaging and characterization.**

For transmission electron microscopy (TEM) imaging, a drop of the liposome dispersion was placed on Cu grid, 150 mesh, coated with an amorphous carbon film. Before sample deposition, each grid was plasma-treated (O^2+^Ar plasma, 15 W, 2 min) to remove hydrocarbon residues from the carbon film. For staining, sample grids were treated with 1% uranyl acetate solution in water. TEM analyses were carried out with a transmission electron microscope (JEOL 1011, Tokyo, JAPAN). Size and Z-potential distributions of a 100 µg/ml LGI1MIM-LSs dispersion were analyzed using a Zetasizer Nano ZSP (Malvern Instrument). To assess the amount of LGI1MIM in LGI1MIM-LSs, samples were prepared for the SDS-PAGE assay. Signal quantification of band intensities (pixel values) was performed using ImageJ software (Wayne Rasband, NIH) (Supplementary Fig. 6a, 6b).

**WST-1 proliferation assay**

Cell proliferation was assessed using WST-1 (2-(4-iodophenyl)-3-(4-nitrophenyl)-5-(2,4-disulfophenyl)-2H-tetrazolium) sodium salt in a premix electrocoupling solution (BioVision). Briefly, T347 cells were cultured for 3 days with media supplemented with vehicle, LSs, LGI1MIM, or LGI1MIM-LS. Samples were then washed with PBS and incubated in phenol-red free DMEM supplemented with 10% FBS and the premix electrocupling solution (1:11 dilution) for 30 min. The medium was then collected and the absorbance of the supernatants was measured at 450 nm with a microplate reader (Victor3, PerkinElmer). The blank was subtracted from the absorbance measurements and values were normalized to the non-treated controls.

**Multicellular blood-brain barrier (BBB) *in vitro* model**

BBB model was obtained by culturing the brain-derived endothelioma bEnd.3 cells (ATCC® CRL-2299^™^ at 8X10^4^ cells/cm^2^) and the C8D1A brain astrocytes (ATCC® CRL-2541™ at 2X10^4^ cells/cm^2^) on the luminal and abluminal side of 3 μm porous transwells (Corning Incorporated). Cells were cultured for 4 days before the transendothelial electric resistance (TEER) was measured using a Millipore Millicell ERS-2 Volt-Ohmmeter.

Immunofluorescence against tight junction marker zonula occludens-1 (ZO-1, Invitrogen) was performed on fixed and permeabilized cells. A subsequent staining of the f-actin and nuclei was carried out using TRITC-conjugated phalloidin (Millipore) and Hoechst (33342, Invitrogen). 3D imaging of the bEnd.3 and C8D1A cells was performed with a confocal laser scanning microscope (CLSM; C2s system, Nikon).

For the evaluation of BBB crossing, the cells in both the luminal and abluminal compartments were incubated with appropriate media, and DiO-stained LGI1MIM-LSs were added in the luminal medium at different concentrations (15, 50, 150 and 500 µg/ml). Subsequently, the fluorescence emission of the abluminal solution was assessed at different time points (10min, 1 hr, 4hr, 24hr) using VICTOR Multilabel Plate Reader (λ_ex_ 485 nm, λ_em_ 535 nm). The blank value was subtracted from all the measurements and the data was converted to concentration by normalizing the fluorescence emission of the DiO-stained LGI1MIM-LS solution at 83 µg/ml concentration which is the maximum concentration that can be reached by this system at equilibrium.

**Evaluation of LGI1MIM-LSs internalization, Ki-67 expression and cell cycle analysis in T347 cells**

Abluminal compartments were transferred to 24 wells plates where T347 cells were seeded 24 h prior to the start of the experiment. 500 µg/ml LGI1MIM-LSs was added to the luminal compartment and incubated for 72 hrs. Liposome uptake, cell viability, Ki-67 expression and cell cycle analysis were investigated in the T347 cells cultured in the abluminal compartment. 3D CLSM of DiO-stained LGI1MIM-LSs internalized in T347 cells was performed using a C2s system (Nikon). Cell viability was carried out using the WST-1 assay.

Ki-67 immunofluorescence was performed on T347 cells. Ki-67 antibody was used for staining with a TRITC-conjugated secondary antibody (Millipore). CLSM imaging was carried out with a C2s system (Nikon) and Ki-67 nuclei count was carried out using NIS-Elements software (Nikon).

Cell cycle was analyzed with the propidium iodide (PI, Sigma) DNA staining as per manufacturer’s instructions. The cell-cycle was measured using a flow cytometer (CytoFLEX Flow Cytometer, Beckman Coulter).

**References**

1 Priedigkeit, N. *et al.* Exome-capture RNA sequencing of decade-old breast cancers and matched decalcified bone metastases. *JCI Insight* **2**, doi:10.1172/jci.insight.95703 (2017).

2 Vareslija, D. *et al.* Transcriptome Characterization of Matched Primary Breast and Brain Metastatic Tumors to Detect Novel Actionable Targets. *J Natl Cancer Inst* **111**, 388-398, doi:10.1093/jnci/djy110 (2019).

3 Leek, J. T. svaseq: removing batch effects and other unwanted noise from sequencing data. *Nucleic Acids Res* **42**, doi:10.1093/nar/gku864 (2014).

4 Love, M. I., Huber, W. & Anders, S. Moderated estimation of fold change and dispersion for RNA-seq data with DESeq2. *Genome Biol* **15**, 550, doi:10.1186/s13059-014-0550-8 (2014).

5 McBryan, J. *et al.* Transcriptomic Profiling of Sequential Tumors from Breast Cancer Patients Provides a Global View of Metastatic Expression Changes Following Endocrine Therapy. *Clin Cancer Res* **21**, 5371-5379, doi:10.1158/1078-0432.ccr-14-2155 (2015).

6 Robinson, M. D., McCarthy, D. J. & Smyth, G. K. edgeR: a Bioconductor package for differential expression analysis of digital gene expression data. *Bioinformatics* **26**, 139-140, doi:10.1093/bioinformatics/btp616 (2010).

7 Yu, G., Wang, L. G., Han, Y. & He, Q. Y. clusterProfiler: an R package for comparing biological themes among gene clusters. *OMICS* **16**, 284-287, doi:10.1089/omi.2011.0118 (2012).

8 Naba, A. *et al.* The extracellular matrix: Tools and insights for the "omics" era. *Matrix Biol* **49**, 10-24, doi:10.1016/j.matbio.2015.06.003 (2016).

9 Cotto, K. C. *et al.* DGIdb 3.0: a redesign and expansion of the drug-gene interaction database. *Nucleic Acids Res* **46**, D1068-D1073, doi:10.1093/nar/gkx1143 (2018).

10 Gu, Z., Eils, R. & Schlesner, M. Complex heatmaps reveal patterns and correlations in multidimensional genomic data. *Bioinformatics* **32**, 2847-2849, doi:10.1093/bioinformatics/btw313 (2016).

11 Yang, J. & Zhang, Y. Protein Structure and Function Prediction Using I-TASSER. *Curr Protoc Bioinformatics* **52**, 5 8 1-15, doi:10.1002/0471250953.bi0508s52 (2015).

12 Kurcinski, M., Jamroz, M., Blaszczyk, M., Kolinski, A. & Kmiecik, S. CABS-dock web server for the flexible docking of peptides to proteins without prior knowledge of the binding site. *Nucleic Acids Res* **43**, W419-424, doi:10.1093/nar/gkv456 (2015).

13 Marino, A. *et al.* Piezoelectric barium titanate nanostimulators for the treatment of glioblastoma multiforme. *J Colloid Interface Sci* **538**, 449-461, doi:10.1016/j.jcis.2018.12.014 (2019).
