# Supplementary material for: ADAM22/LGI1 complex as a new actionable target for breast cancer brain metastasis
Source: BMC Med. 2020 Nov 19;18:349. doi: 10.1186/s12916-020-01806-4 (PMC7677775; doi:10.1186/s12916-020-01806-4)
Supplement: Supplementary file 2 — Additional file 2: Figure S1. Confirmation of LY2 ADAM22 CRISPR knockout cell line. (a) RFP expression confirming CRISPR /Cas9 mediated double stranded break and insertion of an RFP tagged HDR plasmid (b) FACS gating strategy based on high RFP expression to single cell sort CRISPR/Cas9 ADAM22 KO cells (right) from LY2 WT cells (left) (c) Confirmation of ADAM22 gene silencing in an LY2 ADAM22 KO clone (Clone H (C-H)) by PCR and Western blot analysis (n = 4). *** p < 0.0005 (d) Genotyping of Clone H using primers which flank the ADAM22 CRISPR sgRNA sites 1 and 3 (left) and sgRNA 2 (right). Clone H contains a homozygous deletion at cut site 1 & 3 and a heterozygous HDR insertion at cut site 2. (e) Sanger sequencing of the homozygous 58 bp deletion in Clone H (red) flanked by the sgRNA 1 and 3 cut sites (lilac) in LY2 WT cells. Figure S2. Confirmation of LY2 lentiviral ADAM22 knock in (KI) cell line. (a) GFP expression confirming successful transduction of LY2 cells with lentiviral ADAM22 particles. (b) FACS gating strategy based on high GFP expression to purify LY2 ADAM22 KI cells (right) from LY2 WT cells (left). (c) Confirmation of ADAM22 gene overexpression in the LY2 lentiADAM22 KI cell line. *p < 0.05. (d) Western blot confirmation of ADAM22 overexpression in LY2 lentiADAM22 cells versus LY2 WT cells. Figure S3. ADAM22 KO, KI and WT cells. (a) ADAM22 protein expression in each biological replicate used for the RPPA study. (b) IVIS imaging of luciferase activity in LY2 luc, Clone H luc (ADAM22 KO) and lentiA22 luc (ADAM22 KI) cells in vitro after treatment with 15 μg/ml of luciferin. (c) Luciferase activity was comparable across each cell line with respect to cell number. (d) Tumour weight was significantly reduced in LY2 ADAM22 KO tumours versus LY2 WT. Tumour weight ± SEM, Unpaired Mann Whitney two tailed t test *p < 0.05. Figure S4. Quantification of LGIMIM peptide in LSs. (a) Transmission electron microscopy (TEM) imaging of LGI1MIM-LSs and SDS-page stained wit [file 12916_2020_1806_MOESM2_ESM.pptx]

## Slide 1
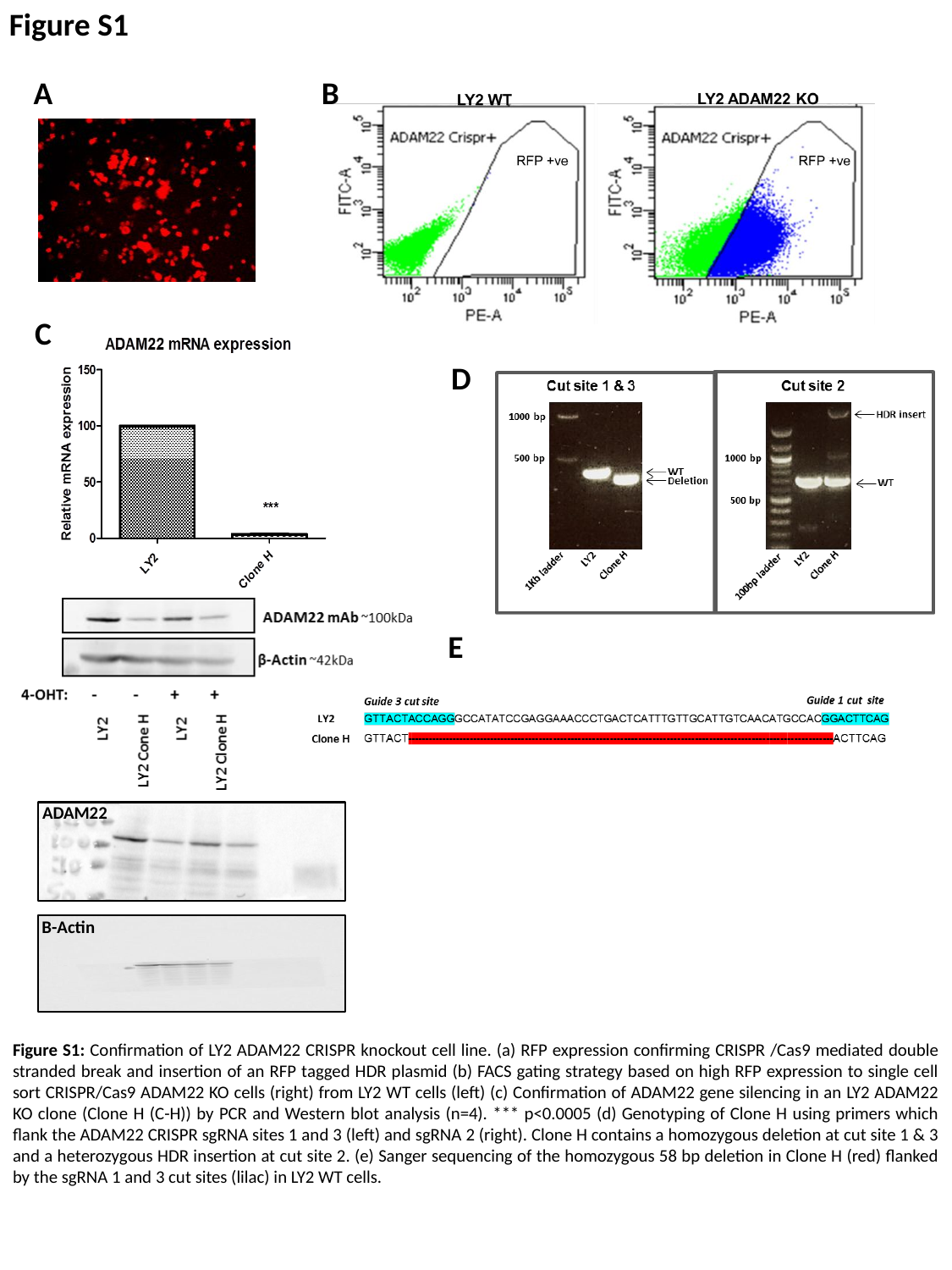

Figure S1
A
B
C
D
E
ADAM22
Β-Actin
Figure S1: Confirmation of LY2 ADAM22 CRISPR knockout cell line. (a) RFP expression confirming CRISPR /Cas9 mediated double stranded break and insertion of an RFP tagged HDR plasmid (b) FACS gating strategy based on high RFP expression to single cell sort CRISPR/Cas9 ADAM22 KO cells (right) from LY2 WT cells (left) (c) Confirmation of ADAM22 gene silencing in an LY2 ADAM22 KO clone (Clone H (C-H)) by PCR and Western blot analysis (n=4). *** p<0.0005 (d) Genotyping of Clone H using primers which flank the ADAM22 CRISPR sgRNA sites 1 and 3 (left) and sgRNA 2 (right). Clone H contains a homozygous deletion at cut site 1 & 3 and a heterozygous HDR insertion at cut site 2. (e) Sanger sequencing of the homozygous 58 bp deletion in Clone H (red) flanked by the sgRNA 1 and 3 cut sites (lilac) in LY2 WT cells.

## Slide 2
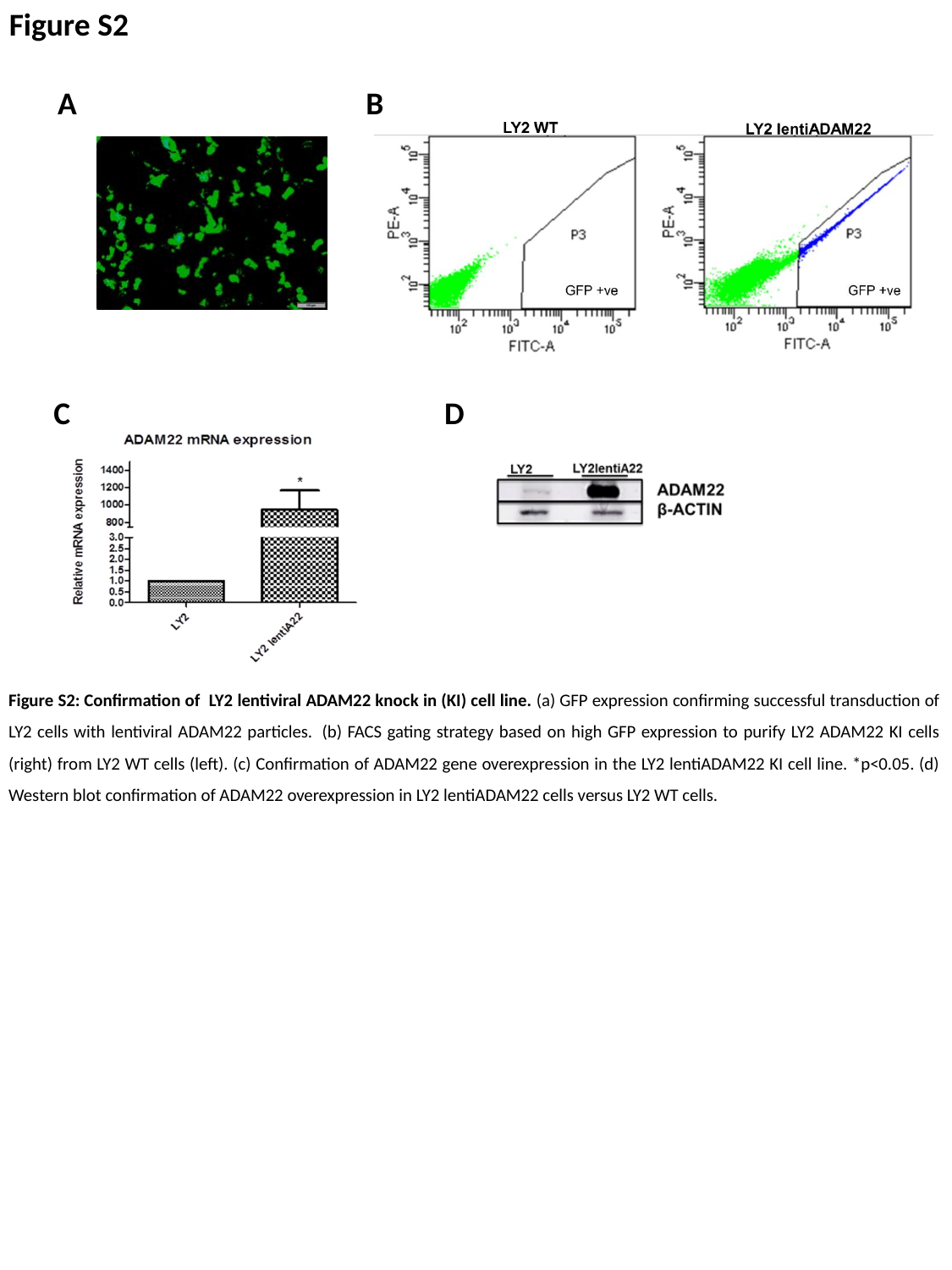

Figure S2
A
B
C
D
Figure S2: Confirmation of LY2 lentiviral ADAM22 knock in (KI) cell line. (a) GFP expression confirming successful transduction of LY2 cells with lentiviral ADAM22 particles.  (b) FACS gating strategy based on high GFP expression to purify LY2 ADAM22 KI cells (right) from LY2 WT cells (left). (c) Confirmation of ADAM22 gene overexpression in the LY2 lentiADAM22 KI cell line. *p<0.05. (d) Western blot confirmation of ADAM22 overexpression in LY2 lentiADAM22 cells versus LY2 WT cells.

## Slide 3
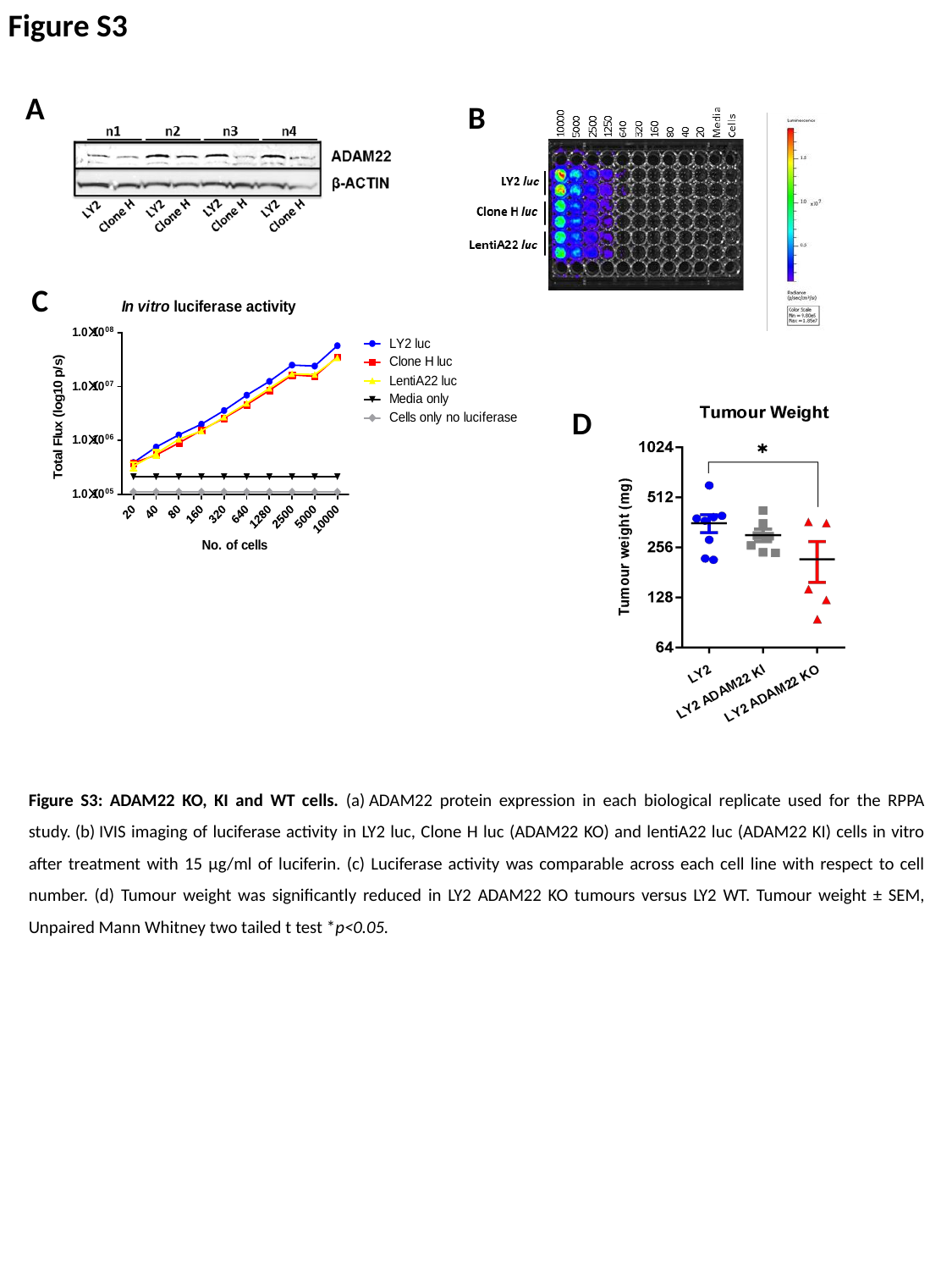

Figure S3
A
B
C
D
Figure S3: ADAM22 KO, KI and WT cells. (a) ADAM22 protein expression in each biological replicate used for the RPPA study. (b) IVIS imaging of luciferase activity in LY2 luc, Clone H luc (ADAM22 KO) and lentiA22 luc (ADAM22 KI) cells in vitro after treatment with 15 µg/ml of luciferin. (c) Luciferase activity was comparable across each cell line with respect to cell number. (d) Tumour weight was significantly reduced in LY2 ADAM22 KO tumours versus LY2 WT. Tumour weight ± SEM, Unpaired Mann Whitney two tailed t test *p<0.05.

## Slide 4
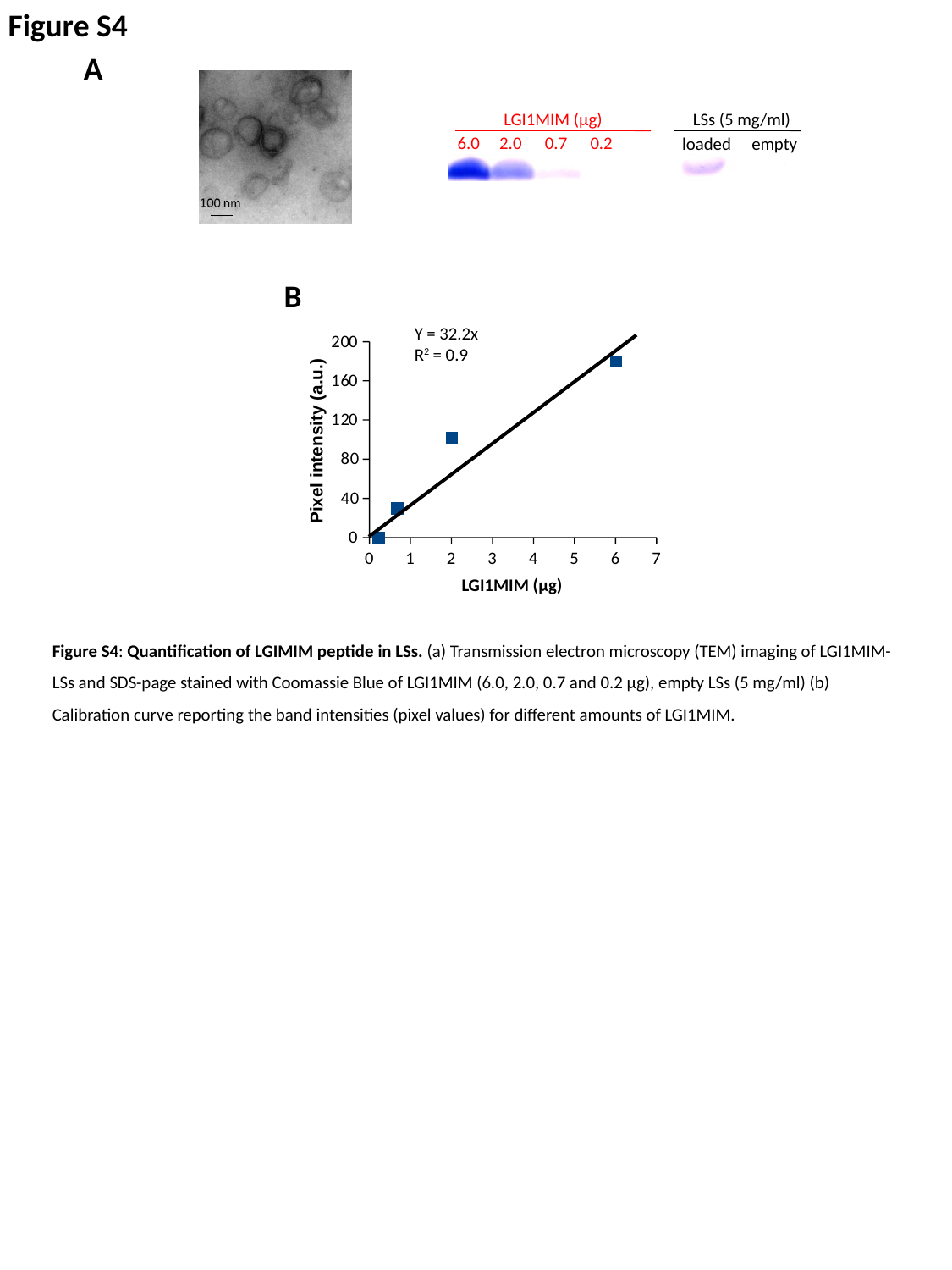

Figure S4
A
LGI1MIM (µg)
LSs (5 mg/ml)
6.0
2.0
0.7
0.2
loaded
empty
B
Y = 32.2x
R2 = 0.9
Pixel intensity (a.u.)
LGI1MIM (µg)
Figure S4: Quantification of LGIMIM peptide in LSs. (a) Transmission electron microscopy (TEM) imaging of LGI1MIM-LSs and SDS-page stained with Coomassie Blue of LGI1MIM (6.0, 2.0, 0.7 and 0.2 µg), empty LSs (5 mg/ml) (b) Calibration curve reporting the band intensities (pixel values) for different amounts of LGI1MIM.

## Slide 5
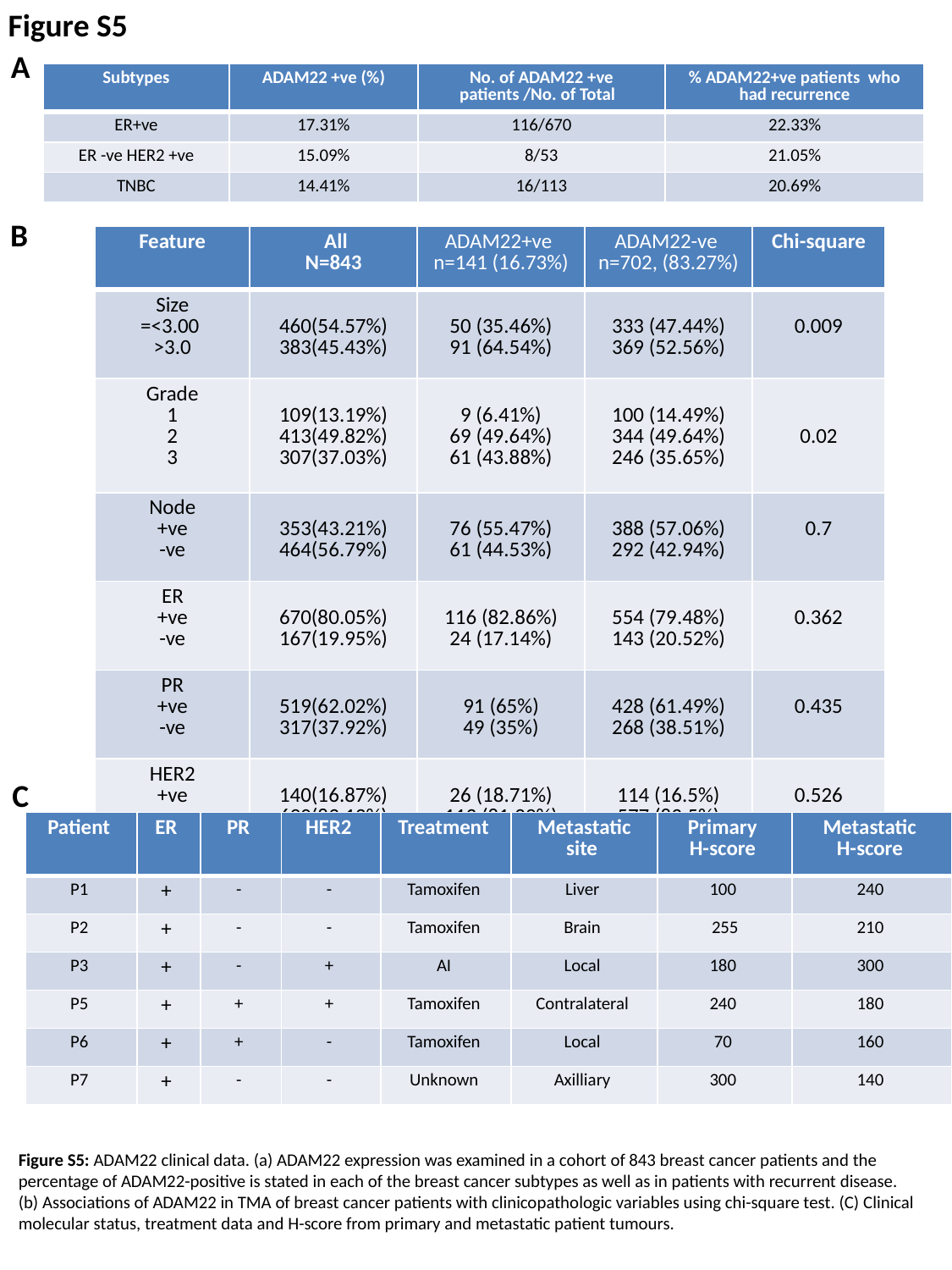

Figure S5
A
| Subtypes | ADAM22 +ve (%) | No. of ADAM22 +ve patients /No. of Total | % ADAM22+ve patients  who had recurrence |
| --- | --- | --- | --- |
| ER+ve | 17.31% | 116/670 | 22.33% |
| ER -ve HER2 +ve | 15.09% | 8/53 | 21.05% |
| TNBC | 14.41% | 16/113 | 20.69% |
B
| Feature​ | All N=843​ | ADAM22+ve  n=141 (16.73%) | ADAM22-ve  n=702, (83.27%) | Chi-square​ |
| --- | --- | --- | --- | --- |
| Size​ =<3.00  >3.0​ | ​ 460(54.57%)​ 383(45.43%)​ | 50 (35.46%) 91 (64.54%) | 333 (47.44%) 369 (52.56%) | ​ 0.009​ |
| Grade​ 1​ 2​ 3​ | ​ 109(13.19%)​ 413(49.82%)​ 307(37.03%)​ | 9 (6.41%) 69 (49.64%) 61 (43.88%) | 100 (14.49%) 344 (49.64%) 246 (35.65%) | ​ ​ 0.02​ |
| Node​ +ve​ -ve​ | ​ 353(43.21%)​ 464(56.79%)​ | 76 (55.47%) 61 (44.53%) | 388 (57.06%) 292 (42.94%) | ​ 0.7​ |
| ER​ +ve​ -ve​ | ​ 670(80.05%)​ 167(19.95%)​ | 116 (82.86%) 24 (17.14%) | 554 (79.48%) 143 (20.52%) | ​ 0.362​ |
| PR​ +ve​ -ve​ | ​ 519(62.02%)​ 317(37.92%)​ | 91 (65%) 49 (35%) | 428 (61.49%) 268 (38.51%) | ​ 0.435​ |
| HER2​ +ve​ -ve​ | ​ 140(16.87%)​ 690(83.13%)​ | 26 (18.71%) 113 (81.29%) | 114 (16.5%) 577 (83.5%) | ​ 0.526​ ​ |
C
| Patient | ER | PR | HER2 | Treatment | Metastatic site | Primary  H-score | Metastatic H-score |
| --- | --- | --- | --- | --- | --- | --- | --- |
| P1 | + | - | - | Tamoxifen | Liver | 100 | 240 |
| P2 | + | - | - | Tamoxifen | Brain | 255 | 210 |
| P3 | + | - | + | AI | Local | 180 | 300 |
| P5 | + | + | + | Tamoxifen | Contralateral | 240 | 180 |
| P6 | + | + | - | Tamoxifen | Local | 70 | 160 |
| P7 | + | - | - | Unknown | Axilliary | 300 | 140 |
Figure S5: ADAM22 clinical data. (a) ADAM22 expression was examined in a cohort of 843 breast cancer patients and the percentage of ADAM22-positive is stated in each of the breast cancer subtypes as well as in patients with recurrent disease. (b) Associations of ADAM22 in TMA of breast cancer patients with clinicopathologic variables using chi-square test. (C) Clinical molecular status, treatment data and H-score from primary and metastatic patient tumours.

## Slide 6
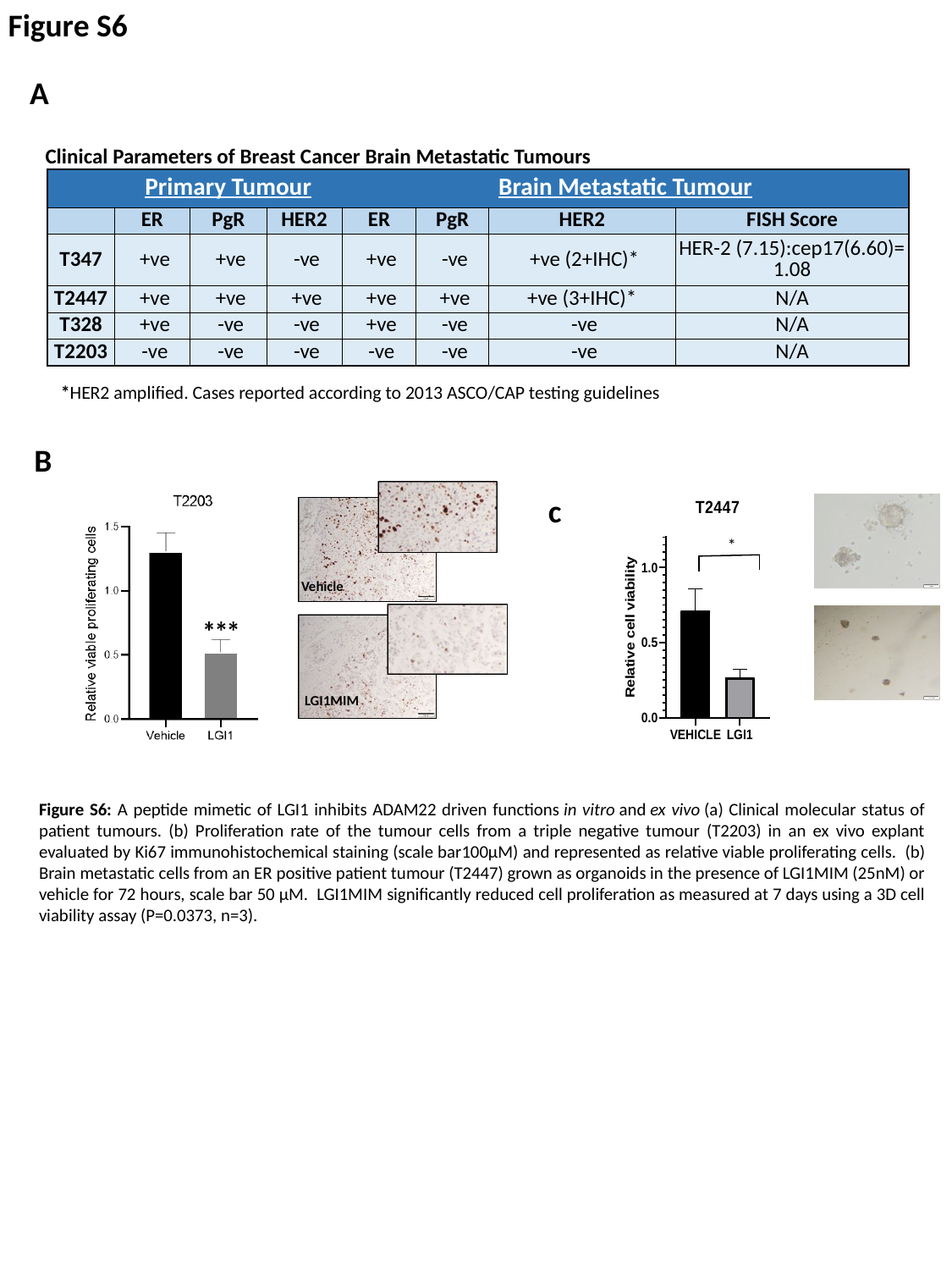

Figure S6
A
Clinical Parameters of Breast Cancer Brain Metastatic Tumours
| | Primary Tumour | | | Brain Metastatic Tumour | | | |
| --- | --- | --- | --- | --- | --- | --- | --- |
| | ER | PgR | HER2 | ER | PgR | HER2 | FISH Score |
| T347 | +ve | +ve | -ve | +ve | -ve | +ve (2+IHC)\* | HER-2 (7.15):cep17(6.60)= 1.08 |
| T2447 | +ve | +ve | +ve | +ve | +ve | +ve (3+IHC)\* | N/A |
| T328 | +ve | -ve | -ve | +ve | -ve | -ve | N/A |
| T2203 | -ve | -ve | -ve | -ve | -ve | -ve | N/A |
*HER2 amplified. Cases reported according to 2013 ASCO/CAP testing guidelines
B
c
*
Vehicle
LGI1MIM
Figure S6: A peptide mimetic of LGI1 inhibits ADAM22 driven functions in vitro and ex vivo (a) Clinical molecular status of patient tumours. (b) Proliferation rate of the tumour cells from a triple negative tumour (T2203) in an ex vivo explant evaluated by Ki67 immunohistochemical staining (scale bar100µM) and represented as relative viable proliferating cells.  (b) Brain metastatic cells from an ER positive patient tumour (T2447) grown as organoids in the presence of LGI1MIM (25nM) or vehicle for 72 hours, scale bar 50 µM.  LGI1MIM significantly reduced cell proliferation as measured at 7 days using a 3D cell viability assay (P=0.0373, n=3).

## Slide 7
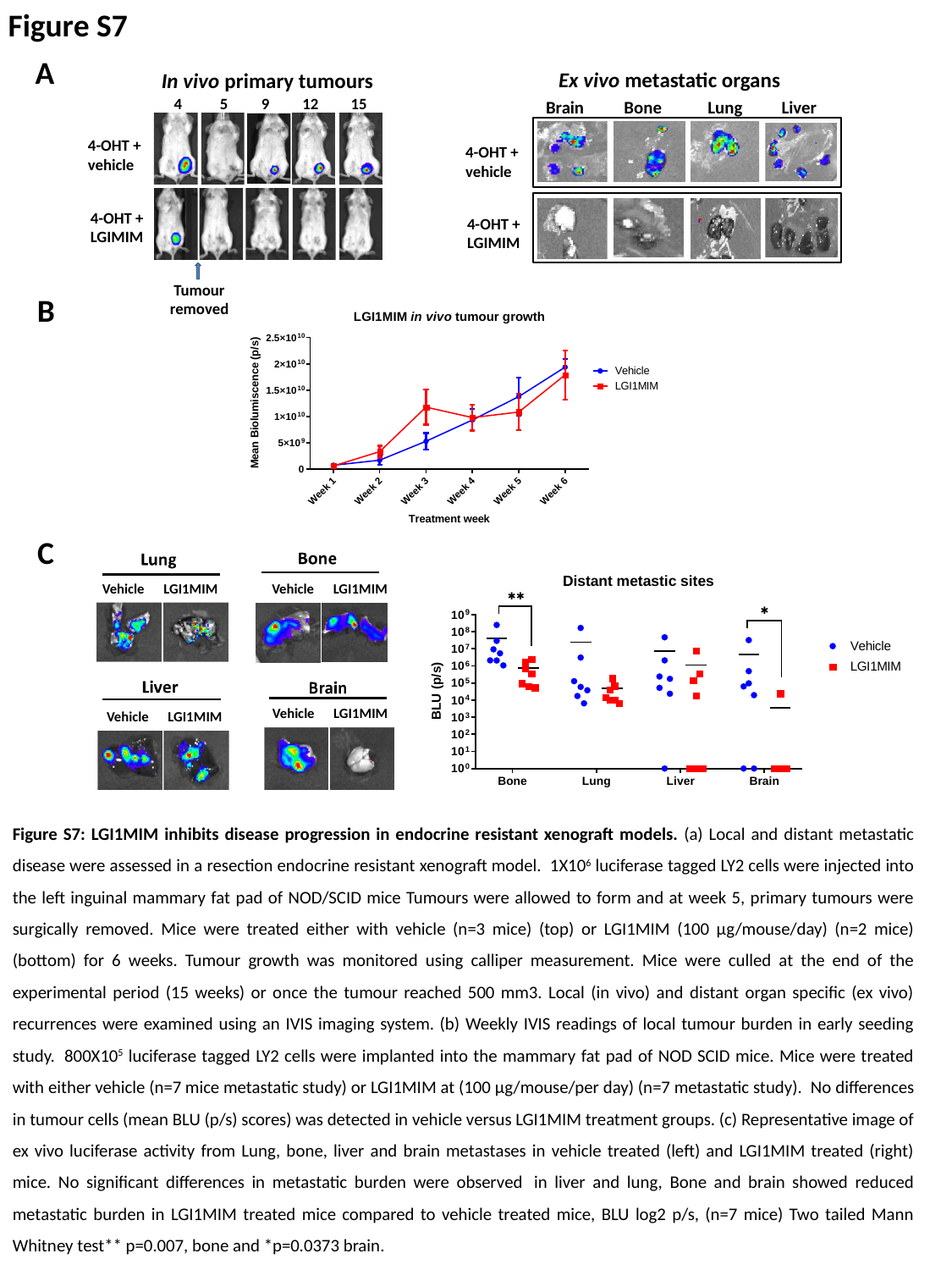

Figure S7
A
Ex vivo metastatic organs
In vivo primary tumours
4
5
9
12
15
4-OHT +
vehicle
4-OHT +
LGIMIM
Tumour
removed
Brain
Bone
Lung
Liver
4-OHT +
vehicle
4-OHT +
LGIMIM
B
C
 Vehicle LGI1MIM
 Vehicle LGI1MIM
 Vehicle LGI1MIM
 Vehicle LGI1MIM
Figure S7: LGI1MIM inhibits disease progression in endocrine resistant xenograft models. (a) Local and distant metastatic disease were assessed in a resection endocrine resistant xenograft model.  1X106 luciferase tagged LY2 cells were injected into the left inguinal mammary fat pad of NOD/SCID mice Tumours were allowed to form and at week 5, primary tumours were surgically removed. Mice were treated either with vehicle (n=3 mice) (top) or LGI1MIM (100 μg/mouse/day) (n=2 mice) (bottom) for 6 weeks. Tumour growth was monitored using calliper measurement. Mice were culled at the end of the experimental period (15 weeks) or once the tumour reached 500 mm3. Local (in vivo) and distant organ specific (ex vivo) recurrences were examined using an IVIS imaging system. (b) Weekly IVIS readings of local tumour burden in early seeding study.  800X105 luciferase tagged LY2 cells were implanted into the mammary fat pad of NOD SCID mice. Mice were treated with either vehicle (n=7 mice metastatic study) or LGI1MIM at (100 µg/mouse/per day) (n=7 metastatic study).  No differences in tumour cells (mean BLU (p/s) scores) was detected in vehicle versus LGI1MIM treatment groups. (c) Representative image of ex vivo luciferase activity from Lung, bone, liver and brain metastases in vehicle treated (left) and LGI1MIM treated (right) mice. No significant differences in metastatic burden were observed  in liver and lung, Bone and brain showed reduced metastatic burden in LGI1MIM treated mice compared to vehicle treated mice, BLU log2 p/s, (n=7 mice) Two tailed Mann Whitney test** p=0.007, bone and *p=0.0373 brain.
